# Carotenoid Accumulation and Its Contribution to Flower Coloration of *Osmanthus fragrans*

**DOI:** 10.3389/fpls.2018.01499

**Published:** 2018-10-16

**Authors:** Yiguang Wang, Chao Zhang, Bin Dong, Jianxin Fu, Shaoqing Hu, Hongbo Zhao

**Affiliations:** ^1^Department of Ornamental Horticulture, School of Landscape Architecture, Zhejiang Agriculture and Forestry University, Hangzhou, China; ^2^College of Civil Engineering and Architecture, Zhejiang Sci-Tech University, Hangzhou, China

**Keywords:** *Osmanthus fragrans*, cultivars, flower color, pigments, carotenoid biosynthesis

## Abstract

Among naturally occurring pigments, carotenoids are importantly involved in the photosynthesis of plants and responsible for the coloration of petals and fruits. *Osmanthus fragrans* Lour., a famous ornamental plant, has many cultivars with different flower color. Petal coloration in *O. fragrans* mainly depends on the kinds of carotenoids and their contents. To investigate the mechanism of flower coloration in different cultivars, an analysis of phenotypic classification, phytochemistry, as well as the expression of carotenoid metabolism genes based on different groups was performed in the present study. Two main clusters including the orange-red cluster containing Aurantiacus cultivars and the yellowish-white cluster containing the other three cultivar groups were classified using the CIE*L^∗^a^∗^b^∗^* system. No significant differences in flavonoid contents were observed between these two clusters. However, carotenoids, especially α-carotene and β-carotene, were found to have crucial roles in the diversity of floral coloration among the different cultivars. Carotenoid compositions in the petals of cultivars from both clusters consisted of α-carotene, β-carotene, α-cryptoxanthin, β-cryptoxanthin, lutein, and zeaxanthin, but carotenoid accumulation patterns during the flowering process were different. The petals of the yellowish-white cultivars exhibited high contents of β-carotene, lutein and α-carotene, whereas the petals of the orange-red cultivars mainly contained β-carotene and α-carotene. The profound diversity in the total carotenoid concentrations in the two clusters was determined by the transcript levels of *OfCCD4*. Furthermore, the accumulation of upstream products with orange color in orange-red cultivars was partially due to the low expression of *OfCHYB*, whereas the relatively higher *OfCHYB* expression in the petals of the yellowish-white cultivars led to higher proportions of lutein, which is yellow. We also found that downregulation of *OfLCYE*, which encodes 𝜀-ring cyclase, indicated that the carotenoid flux of most cultivars mainly resulted in more β, β-branched products. Additionally, carotenoid biosynthesis in green tissues and petals was compared, revealing the tissue specificity of carotenoid accumulation in *O. fragrans*. Therefore, the effects of multiple genes on carotenoid accumulation give rise to the colorful *O. fragrans*.

## Introduction

Flower color is one of the most important traits of ornamental plants and is attributed to various pigments that can be divided into three major classes including flavonoids, carotenoids, and betalains (Grotewold, 2006). Among these pigments, carotenoids are responsible for the colors ranging from yellow to red (Tanaka et al., 2008).

Carotenoids, a group of hydrophobic pigments, are stored in differentiated plastids. In the chloroplasts of green tissues, carotenoids are essential in photosynthesis for functions such as photosystem assembly, light harvesting, and photoprotection (Domonkos et al., 2013), while non-green tissues, such as flowers, fruits and seeds, accumulate carotenoids in chromoplasts and display vivid colors. Carotenoids are precursors for vitamin A and play important roles not only in plants but also in human health (Asensi-Fabado and Munné-Bosch, 2010). More than 750 naturally occurring carotenoids which are mainly divided into carotenes and xanthophylls, have been identified from plants, animals, and microorganisms (Nisar et al., 2015). The carotenoid profiles in green tissues of most plant are similar, whereas those in non-green tissues are distinct and vary considerably depending on the plants species (Ohmiya, 2013; Yuan et al., 2015). Therefore, different regulatory mechanisms for carotenoid accumulation exist in various plant species or their tissues.

**FIGURE 1:**
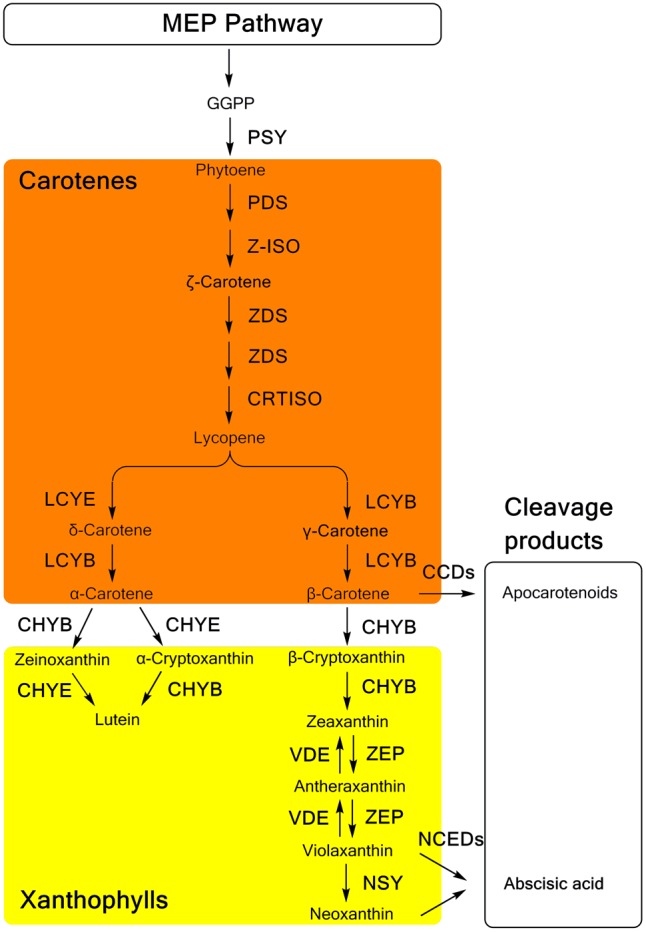
The carotenoid biosynthesis pathway in plants. MEP methylerythritol phosphate; GGPP, geranylgeranyl diphosphate; PSY, phytoene synthase; PDS, phytoene desaturase; Z-ISO, ζ-carotene isomerase; ZDS, ζ-carotene desaturase; CRTISO, carotenoid isomerase; LCYB, lycopene β-ring cyclase; LCYE, lycopene 𝜀-ring cyclase; CHYB, β-ring hydroxylase; CHYE, 𝜀-ring hydroxylase; ZEP, zeaxanthin epoxidase; VDE, violaxanthin de-epoxidase; NSY, neoxanthin synthase; CCDs, carotenoid cleavage dioxygenases; NCED, 9-*cis*-epoxycarotenoid dioxygenase.

Carotenoid biosynthesis (**Figure 1**), as well as the related genes and enzymes, have been well elucidated in many plants (Nisar et al., 2015; Yuan et al., 2015). Generally, as a part of the terpenoid biosynthesis pathway, carotenoid biosynthesis originates from the condensation of isopentenyl diphosphate (IPP) and dimethylallyl diphosphate (DMAPP), which are generated in the methylerythritol phosphate (MEP) pathway (Rodríguez-Concepción, 2010). Through the catalysis of phytoene synthase (PSY), the first C40 carotenoid 15-*cis*-phytoene is synthesized with the head-to-head condensation of two molecules of C20 geranylgeranyl diphosphate (GGPP), which are produced from three IPP molecules and one DMAPP molecule. Colorless phytoene is converted into all-*trans*-lycopene, which has a bright red color, and then catalyzed by two dehydrogenases, phytoene desaturase (PDS) and ζ-carotene desaturase (ZDS), and two isomerases, ζ-carotene isomerase (Z-ISO) and carotenoid isomerase (CRTISO), respectively. Cyclization of lycopene is the branch point of carotenoid synthesis, in which orange α-carotene and β-carotene are generated through catalysis by 𝜀-ring cyclase (LCYE) or β-ring cyclase (LCYB). As precursor substances of xanthophylls, α-carotene and β-carotene are further hydroxylated by different types of hydroxylases. A P450-type 𝜀-ring hydroxylase (CHYE/CYP97C) and a P450-type β-ring hydroxylase (CHYB/CYP97A) are involved in the hydroxylation of α-carotene, yielding yellow lutein. In the β, β-branch of the carotenoid biosynthesis pathway, β-carotene is converted into β-cryptoxanthin and zeaxanthin sequentially through two hydroxylation reactions catalyzed by CHYB. Then, one β-ring of zeaxanthin is epoxidized by zeaxanthin epoxidase (ZEP), yielding antheraxanthin, which can be converted into violaxanthin. However, these two steps of epoxidation are reversible with the effect of violaxanthin de-epoxidase (VDE). Alternatively, violaxanthin can be catalyzed by neoxanthin synthase (NSY) to yield neoxanthin as the product in the final step of the β, β-branch.

In the catabolism pathway, carotenoids are enzymatically cleaved into various types of apocarotenoids (**Figure 1**). The 9-*cis*-epoxycarotenoid dioxygenases (NCEDs), which include five members in *Arabidopsis* specifically cleave 9-*cis*-violaxanthin and 9-*cis*-neoxanthin to yield the ABA precursor, xanthoxin (Tan et al., 2003). Distinctively, a broad range of carotenoids can be cleaved as substrates of carotenoid cleavage dioxygenases (CCDs). Among five CCDs, CCD1, CCD2 and CCD4 are involved in the production of apocarotenoid-derived pigments, flavors, and aromas (Huang et al., 2009; Baldermann et al., 2010; Ma et al., 2013; Cazzonelli, 2014; Frusciante et al., 2014), while CCD7 and CCD8 are related to the synthesis of strigolactones (Seto et al., 2014).

*Osmanthus fragrans* Lour., a famous ornamental tree, is appreciated for its pleasant scent, esthetic value, and edible flowers. Many *O. fragrans* cultivars have a long history of cultivation. In general, four groups including the Albus Group, Luteus Group, Aurantiacus Group, and Asiaticus Group are used to classifying these cultivars, mainly according to the flowering season and flower color (Xiang and Liu, 2007). According to previous research, both the flavonoid and carotenoid metabolism occurs in *O. fragrans* flowers (Mu et al., 2014). Different carotenoid composition and concentrations were found in the petals of ‘Zi Yingui’ (an Albus Group cultivar), ‘Jingui’ (a Luteus Group cultivar), and ‘Chenghong Dangui’ (an Aurantiacus Group cultivar) (Han et al., 2014). Furthermore, some genes related to carotenoid metabolism, including *OfPSY, OfPDS, OfZDS, OfLCYE, OfLCYB, OfHYB, OfZEP, OfNCED, OfCCD1*, and *OfCCD4*, in the petals of *O. fragrans* have been isolated and used for reverse transcription-polymerase chain reaction (RT-PCR) analysis (Huang et al., 2009; Baldermann et al., 2010; Han et al., 2013, 2014). Differential expression of the genes involved in downstream carotenoid synthesis and degradation, e.g., *OfCCD4*, has been suggested to be involved in the different carotenoid accumulation patterns of these three cultivars (Han et al., 2014). Additionally, abundant flavonoids have also been reported to exist in the petals of *O. fragrans* (Tsai et al., 2008; Hung et al., 2012; Chen et al., 2015), and studies on their metabolism regulation have been initiated by some researchers (Mu et al., 2014; Han et al., 2015). However, the contribution of flavonoid concentrations to the petal color of *O. fragrans* remains to be investigated.

Although previous literature has investigated carotenoids and expression of the related genes in *Osmanthus fragrans*, only one cultivar of each cultivar group in the previous research was used (Han et al., 2014). As we know, there are many cultivars of each cultivar group (Albus Group, Luteus Group, Aurantiacus Group, and Asiaticus Group), which have different ornamental trait including flower color. In our research, we used much more cultivars (24 cultivars, in total) from four groups; more carotenoid components were identified by high-performance liquid chromatography-atmospheric pressure chemical ionization-mass spectrometry (HPLC-APCI-MS) combined with authentic standards; and the expression analysis of key carotenogenic genes were selected from transcriptome sequencing of our previous research (Zhang et al., 2016). We also compared the contribution of carotenoids and flavonoids to flower coloration. Additionally, the carotenoid carotenogenic pathways in different tissues were compared to demonstrate the tissue-specific accumulation of carotenoids, which has not been reported. Eventually, we can systematically studied the relationship between the gene expression, carotenoid composition and the final phenotypes of flower color. Therefore, a better understanding of the mechanisms regulating coloration of *O. fragrans* flowers will be achieved.

## Materials and Methods

### Plant Materials

Twenty-four cultivars, belonging to the four cultivar groups of *O. fragrans* (**Table 1**) were selected for this study. Under identical planting conditions, these cultivars were grown in the germplasm repository for *O. fragrans* (located at 30°15′ 23″ N/ 119°43′ 37″ E) at Zhejiang Agriculture and Forestry University, Lin’an, China. The petals of those cultivars were collected at four developmental stages, including the linggeng stage (S1), initial flowering stage (S2), full flowering stage (S3), and late full flowering stage (S4) (Xiang and Liu, 2007) during the flowering period in September, 2015. In addition, different tissues of ‘Yanhong Gui,’ including young leaves (YL), mature leaves (ML), stems (S), flower buds (B) before S1 and petals (P) at S3 were also collected for further investigation. At least three biologic replicates of each cultivar were obtained and stored at -80°C. All samples were divided into two portions, including one portion that was dried for pigment analysis and another portion that was immediately frozen with liquid nitrogen for gene expression analysis.

**Table 1:** The parameters of flower color phynotype of the 24 *O. fragrans* cultivars at full flowering stage.

Sample NO.^a^	Cultivars	RHSCC	CIE*L^∗^a^∗^b^∗^* coordinate ^b^					Content (μg/g, DW) ^c^	
			*L^∗^*	*a^∗^*	*b^∗^*	*C^∗^*	*h*	TC	TF
A1	‘Xiaoye Sugui’	7B	82.58	–0.42	51.56	51.56	–1.56	58.25	121699.15
A2	‘Zaoyin Gui’	4B4C	83.36	0.44	36.46	36.47	0.30	63.98	146882.57
A3	‘Yu Linglong’	9A	82.52	2.00	59.78	59.81	1.54	41.36	136464.86
A4	‘Wanyin Gui’	13A13B	76.82	13.34	43.20	45.24	1.27	113.18	121446.44
A5	‘Jiulong Gui’	3D4C	83.78	–1.10	35.44	35.46	–1.54	22.94	136687.44
A6	‘ZJNL-1’	6A6B	82.70	0.30	52.02	52.02	0.94	93.69	81929.40
B1	‘Yuanban Jingui’	13B	78.78	8.34	51.48	52.16	1.41	77.92	131221.35
B2	‘Hangzhou Huang’	7A7B	79.22	5.00	51.94	52.18	1.47	42.16	131861.13
B3	‘Jinqiu Gui’	15B	77.42	11.96	52.10	53.47	1.34	91.90	128406.90
B4	‘Liuye Jingui’	8B8C	83.50	–1.28	39.20	39.23	–1.54	29.50	106370.27
C1	‘Wuyi Dangui’	28A	64.66	37.22	34.92	51.05	0.75	5469.82	113004.01
C2	‘Mantiao Hong’	N30C	58.66	46.38	44.96	64.61	0.77	10392.69	106380.81
C3	‘Xionghuang’	N25B	63.78	38.96	46.08	60.35	0.87	3441.96	104520.67
C4	‘Zhuangyuan Hong’	N30C	58.52	47.74	43.76	64.76	0.74	9293.47	111360.40
C5	‘Chenghong Dangui’	30C	60.76	45.64	46.46	65.15	0.79	8022.29	114087.32
C6	‘Zhusha Dangui’	N25A	58.58	45.82	45.72	64.75	0.78	6620.17	103333.33
C7	‘Yanhong Gui’	N25B	66.50	35.50	38.16	52.21	0.82	2521.70	99399.23
C8	‘Yingye Dangui’	23A	71.84	25.54	50.60	56.69	1.10	593.91	137980.11
D1	‘Tianxiang Taige’	7B7C	81.72	1.44	49.46	49.49	0.91	39.23	130844.07
D2	‘Foding Zhu’	3C	84.82	–3.56	47.96	48.10	–1.50	31.40	113981.79
D3	‘Sijigui’	5C	83.34	–1.32	49.62	49.64	–1.54	29.72	132941.73
D4	‘Tiannu Sanhua’	8A	82.42	1.26	49.14	49.16	1.55	79.70	134407.06
D5	‘Rixiang Gui’	8B	83.14	–0.60	44.86	44.87	–1.56	40.61	126458.33
D6	‘Chenghuang Sijigui’	5C	82.58	–0.16	50.46	50.47	0.32	33.95	142853.49

*^*a*^A indicates Albus Group; B indicates Luteus Group; C indicates Aurantiacus Group; D indicates Asiaticus Group. ^*b*^The data were expressed by means of five replicates. ^*c*^The data were expressed by means of three replicates.*

### Flower Color Measurement

The colors of petals at the full flowering stage (S3) were described using the Royal Horticultural Society Color Chart (RHSCC) with white paper as the background under the same light condition. In addition, a Minolta CR-10 portable colorimeter (Konica Minolta, Japan) was used to measure the color parameters of these flowers. Parameters of the CIE*L^∗^a^∗^b^∗^* color coordinate including lightness (*L^∗^*) and two chromatic components *a^∗^* and *b^∗^* were measured. Chroma *C^∗^* and the hue angle *h* were calculated according to the equation: *C^∗^* = (*a^∗^*^2^ + *b^∗^*^2^)^1/2^, *h* = arctan (*b^∗^*/*a^∗^*) (Gonnet, 1998). Five measurements of each cultivar were used as replicates.

### Quantitative Determination of Flavonoids

Flavonoids in petals of *O. fragrans* at the full flowering stage (S3) were extracted with 70% methanol (containing 0.1% HCL) in darkness at 4°C for 16 h, and then the extracts were placed in an ultrasonic cleaner for 30 min. The mixture was centrifuged at 12,000 rpm for 5 min at 4°C. The supernatant was collected and filtered through a 0.22-μm micropore prior to HPLC-DAD and HPLC-MS analyses. The experiment was repeated three times for each cultivar.

The quantitative analysis of flavonoids was performed on the same Shimadzu HPLC system used for the carotenoid analysis. A C18 column of Inertsil ODS SP (4.6 mm × 250 mm, 5 μm, Shimadzu GL, Shanghai, China) was used to separate the individual flavonoids with mobile phase A (double distilled water containing 0.1% formic acid) and mobile phase B (acetonitrile containing 0.1% formic acid). The elution gradient was as follows: 0 min, 15% B; 9 min, 30% B; 20 min, 33% B; 37 min, 49% B; 40 min, 49% B; 43 min, 15% B; 50 min, 15% B. Then, 10 μL of the sample was injected for HPLC-DAD analysis. The flow rate was 0.8 mL/min, and the column temperature was set at 35°C. Flavonoids at 350 nm were detected, and UV spectra were scanned in the range of 190–800 nm. The total flavonoid content was defined as the sum of the contents of all flavonoid components, which was calculated based on a linear regression of rutin (*y* = 16250x + 57987, *r*^2^ = 0.9993) under the same chromatographic condition.

### Quantitative Determination of Carotenoids

Extraction of total carotenoids was carried out following the method described by Delpino-Rius et al. (2014) with some modifications. Briefly, the first extraction of powdered samples was performed with methanol, and then hexane and a solution of NaCl (10%, w/v) were added in order. The mixture was continuously shaken until the plant material was colorless. Pooled organic phases were dried under nitrogen stream and saponified overnight using a 6% KOH methanolic solution. The carotenoids were subsequently reextracted with hexane: diethyl ether (3:1, v/v) repeatedly until the aqueous phase became colorless. The organic layers were combined and the solvent was removed under nitrogen stream. Extraction from sample of each cultivar was carried out in triplicate. Notably, all steps were performed in a dark room to avoid degradation and isomerization of carotenoids. Before HPLC-DAD and HPLC-MS analyses, the samples were dissolved in 2 mL of MTBE (methyl *tert-butyl* ether) with 50 μg/ml of the internal standard β-*apo*-8′-carotenal and filtered through a 0.22-μm micropore.

Carotenoids were quantified on a Shimadzu HPLC system (Kyoto, Japan) equipped with an LC-20AT pump, an SPD-M20A DAD detector, a CTO-10AS VP column oven, and an SIL-20A auto injector. Carotenoids were separated on a column of Inertsil C30 S-Selec (4.6 × 250 mm, 5 μm, Shimadzu GL, Shanghai, China) at a column temperature of 25°C and a flow rate of 0.8 mL/min. The injection volume of the filtered sample was 10 μL. The mobile phases consisted of solvent A (methanol) and solvent B (MTBE). The gradient elution program was set as follows: 0 min, 0% B; 18 min, 46% B; 35 min, 70% B; 37 min, 70% B; 40 min, 0% B; 47 min, 0% B. UV spectra were recorded from 190 to 800 nm, and carotenoids were analyzed at 450 nm. The contents of each carotenoid and total carotenoids were calculated according to the peak area ratios with β-*apo*-8′-carotenal.

### Identification of Carotenoid Components

The major carotenoids were identified using an Agilent-1200 HPLC system equipped with a 6210 time-of-flight mass spectrometer (Agilent Technologies, Palo Alto, CA, United States). Atmospheric pressure chemical ionization (ApCI) was applied in positive ion mode for MS. The column, solvents, and gradient elution procedure were the same as those used in the HPLC-DAD analysis described above. The MS conditions were as follows: High purity nitrogen (99.999%) was used as a nebulizing (60 psi) and drying gas at a flow rate of 6 L/min. The vaporizer temperature and drying gas temperature were set at 450 and 300°C, respectively. Other parameters included a fragmentor voltage of 175 V, a skimmer voltage of 6 V, a capillary voltage of 3.5 kV and a corona current of 4 μA. The scan range was set from 100 to 1000 amu. The carotenoid components were preliminarily identified according to the mass spectrometry (MS) fragments and the UV-visible spectrum, including maximum absorption wavelengths (λmax) and spectral fine structure (%III/II). The final identification was carried out by comparison with reference data from authentic standards.

### RNA Extraction and cDNA Synthesis

Extraction of total RNA from all samples was conducted using the RNAprep Pure Plant Kit (Tiangen, China). The RNA concentration and quality were measured on a 2100 Bioanalyzer RNA Nanochip (Agilent, Santa Clara, CA, United States), and then all RNA samples were adjusted to the same concentration. Additionally, the quality of the RNA was further verified using 1.5% (w/v) agarose gel electrophoresis and ethidium bromide staining. One microgram of total RNA was used to synthesize first-strand cDNA with the Reverse Transcriptase M-MLV (Takara, Dalian, China) according to the manufacturer’s protocol.

### Quantitative Real-Time PCR Analysis

Quantitative real-time PCR analysis was performed using the Light Cycler 480 II (Roche, Switzerland). Sixteen genes related to carotenoid metabolism were selected from our previous study (Zhang et al., 2016). The primers for these genes and the *OfACT* gene (as an internal control) are shown in **Supplementary Table S1**. The reaction mixture (20 μL total volume) contained 10 μL of SYBR Premix Ex Taq (Takara, Dalian, China), 2 μL of diluted cDNA, 0.8 μL of each primer (10 μM) and 6.4 μL of ddH2O. The PCR program was carried out with an initial step of 95°C for 30 s and 40 cycles of 95°C for 5 s and 60°C for 30 s, followed by 95°C for 15 s, 60°C for 1 min and 95°C for 15 s for the dissociation stage. The reaction for each sample was performed in triplicate. The value of Cq was calculated by Light Cycler 480 software. The relative expression levels of the target genes were normalized to the relative expression level of *OfACT* using the 2^-ΔCt^ method (Schmittgen and Livak, 2008).

### Statistical Analysis

Hierarchical cluster analysis (HCA) with the between-group linkage method was used to classify different cultivars based on the parameters of flower color. Independent Sample *T* test and Least Significant Difference test were used to calculate statistical significance in **Figures 4, 7**, respectively. The relationships between the parameters of flower color and pigment content were determined using Pearson correlation analysis and multiple linear regression (MLR) which was carried out in a stepwise manner. In addition, Pearson correlation analysis was also used to investigate the relationships between the total carotenoid content and the relative expression levels of related genes at different flowering stages. Each statistical analysis was performed using IBM SPSS Statistics 19.0 (IBM, Armonk, NY, United States).

## Results

### Distributions of the 24 Cultivars Based on Flower Color

**FIGURE 2:**
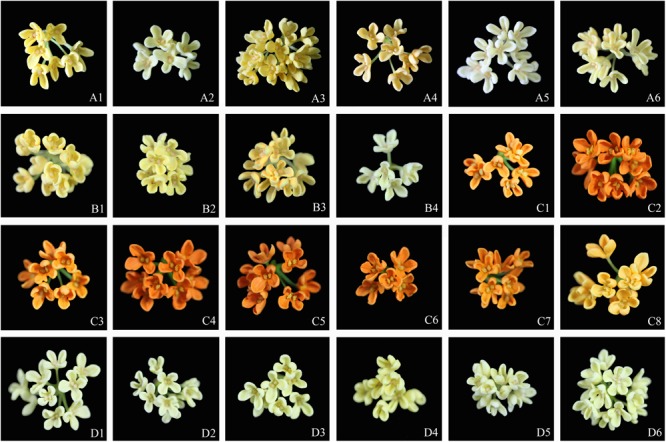
Flowers of the 24 *O. fragrance* cultivars: the Albus Group **(A1–A6)**; Luteus Group **(B1–B4)**; the Aurantiacus Group **(C1–C8)**; the Asiaticus Group **(D1–D6)**. The sample numbers are same as those in **Table 1**.

**FIGURE 3:**
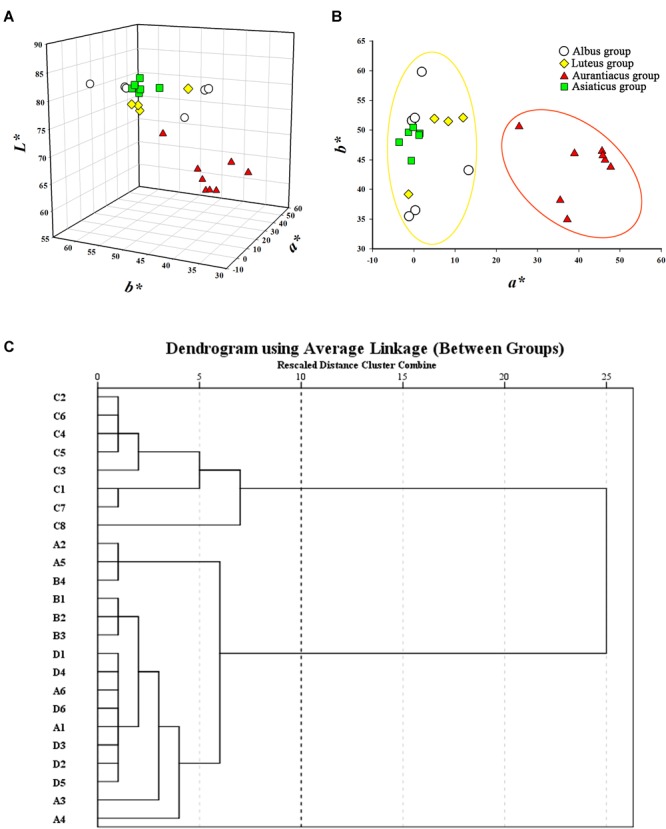
Classification for the 24 *O. fragrans* cultivars based on flower color. **(A)** Flower color distribution of the 24 cultivars based on *a^∗^, b^∗^*, and *L^∗^* values. **(B)** Flower color distribution of the 24 cultivars based on bivariate *a^∗^* and *b^∗^* values. **(C)** Dendrogram of hierarchical cluster for the 24 cultivars based on *L^∗^, a^∗^, b^∗^*, C*^∗^*, and *h*. The abscissa expresses the Euclidean distances and the ordinate denotes the sample numbers.

The flower colors of the 24 cultivars ranged from pale yellow to orange-red (**Figure 2**). The parameters of flower color are listed in **Table 1**. The RHSCC values of the Albus Group (3D-13A), Luteus Group (8C-13B) and Asiaticus Group (3C-8A) intersected, and the RHSCC values of the Aurantiacus Group, which ranged from 23A to N30C, were substantially higher than those of the other cultivar groups. HCA based on values of *L^∗^, a^∗^, b^∗^, C^∗^* and *h* was performed to classify these cultivars. **Figure 3C** shows a dendrogram of the HCA of 24 the cultivars using the between-group linkage method. As the Euclidean distance was 10, the cultivars were divided into two clusters. The first cluster included all cultivars from the Aurantiacus Group, which was defined as the orange-red cluster, whereas the other three cultivar groups constituted the second cluster, which was defined as the yellowish-white cluster. When the Euclidean distance was between 0 and 5, these cultivars were divided more specifically. For instance, in the orange-red cluster, ‘Yingye Dangui’ (C8), which has a lighter orange color was alone in one class. Similarly, ‘Wanyin Gui’ (A4) from the yellowish-white cluster was in its own class due to its deeper yellow color.

The distributions of these two clusters based on CIE*L^∗^a^∗^b^∗^* color coordinates were distinctly different. **Figure 3A** shows that the *L^∗^* values (76.82–84.82) of the yellowish-white cluster were considerably higher than those (58.52–71.84) of the orange-red cluster. In addition, due to significantly higher *a^∗^* values (25.54–47.74; the *a^∗^* values in the yellowish-white cluster were in range of -3.56 to 13.34) in the orange-red cluster, the two clusters were clearly distinguishable in the two-dimensional quadrant of *a^∗^* and *b^∗^* (**Figure 3B**).

### Pigment Contents and the Corresponding Relationships With Flower Color

**FIGURE 4:**
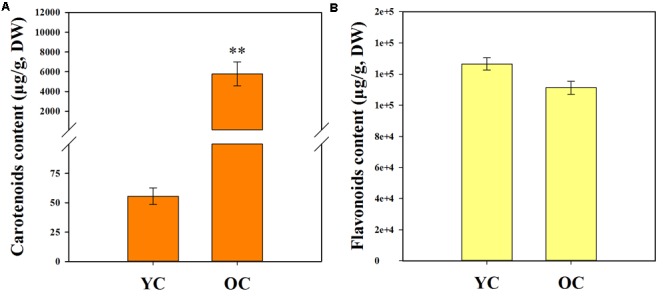
Total carotenoid content **(A)** and total flavonoid content **(B)** in the petals of two color clusters. YC, yellowish-white cluster; OC, orange-red cluster. Data were shown as mean ± SE which were calculated based on the measurements from cluster members. ^∗∗^Denotes significant difference (*P* < 0.01, by *T*-test) between two clusters.

The contents of total carotenoids (TCs) and total flavonoids (TFs) are listed in **Table 1**. Comparison of the pigment contents in the petals of different cultivars revealed that the total carotenoid contents in the petals of cultivars from the orange-red cluster (5794.50 ± 1213.23 μg/g, DW) were much higher than those of the yellowish-white cluster (55.59 ± 6.95 μg/g, DW) (**Figure 4A**). However, differences in the total flavonoid contents between the yellowish-white cluster (126528.50 ± 3905.42 μg/g, DW) and the orange-red cluster (111258.23 ± 4221.00 μg/g, DW) were not remarkable (**Figure 4B**).

To investigate the relationship between flower coloration and the pigment constituents of *O. fragrans*, a Pearson correlation analysis was performed in all *O. fragrans* cultivars, including the yellowish-white cultivars (A1–A6, B1–B4, and D1–D6) and the orange-red cultivars (C1–C8) (**Table 2**). The results showed significant correlations at the 0.01 level between TCs and the values of RHSCC, *L^∗^* and *a^∗^* in combinations of all cultivars, the yellowish-white cultivars and the orange-red cultivars. Among these parameters, the value of *L^∗^* was negatively correlated with TCs, whereas the value of *a^∗^* was positively affected by the TC content. The RHSCC number based on flower color tended to increase while TC increased (**Table 2**). For instance, the petals of cultivars including ‘Mantiao Hong’ (C2), ‘Zhuangyuan Hong’ (C4), ‘Chenghong Dangui’ (C5) and ‘Zhusha Dangui’ (C6), which had the lowest value of *L^∗^* and the highest value of *a^∗^*, contained the highest concentrations of TCs. In addition, the TC content had a positive effect on the value of *C^∗^* when all cultivars were evaluated together. In contrast, although significant correlations at the 0.05 level were observed between the TF content and the values of RHSCC, *L^∗^* and *a^∗^*, the color parameters of the cultivars within the two clusters were not affected by TFs, indicating that the effect of the TF content on the flower color of *O. fragrans* is less important.

**Table 2:** The Pearson correlation between parameters of flower color and content of pigments at full flowering stage.

CIELab coordinate	All cultivars		Yellowish-white cluster		Orange-red cluster	
	TCs	TFs	TCs	TFs	TCs	TFs
RHSCC	**0.862^∗∗^**	**–0.456^∗^**	**0.659^∗∗^**	–0.027	**0.899^∗∗^**	–0.245
*L^∗^*	**–0.918^∗∗^**	**0.489^∗^**	**–0.686^∗∗^**	–0.073	**–0.914^∗∗^**	0.611
*a^∗^*	**0.901^∗∗^**	**–0.479^∗^**	**0.740^∗∗^**	0.089	**0.916^∗∗^**	–0.617
*b^∗^*	–0.267	0.175	0.146	–0.085	–0.056	0.490
*C^∗^*	**0.713^∗∗^**	–0.374	0.209	–0.087	0.690	–0.120
*h/^O^*	0.224	–0.017	**0.613^∗^**	0.135	**–0.759^∗^**	**0.773^∗^**

*^∗^Correlation is significant at the 0.05 level. ^∗∗^Correlation is significant at the 0.01 level.*

### Carotenoid Components and Their Contribution to Flower Color

**FIGURE 5:**
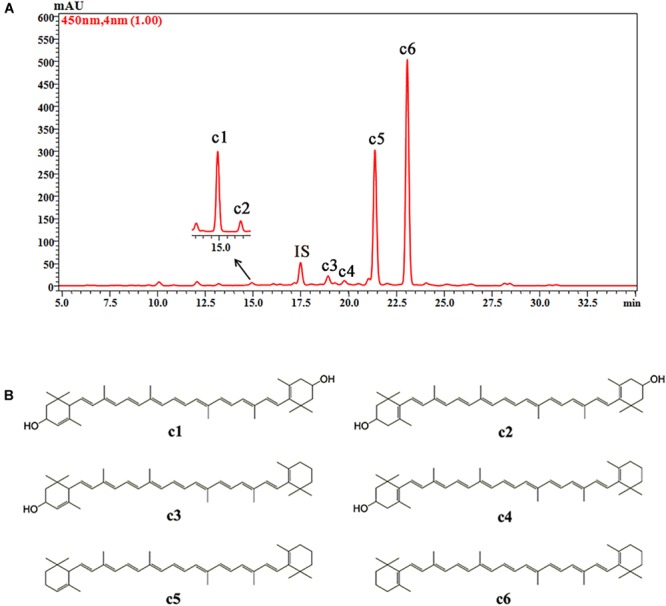
The HPLC chromatogram of carotenoids from petals of *O. fragrans* obtained at 450 nm **(A)** and their chemical structures **(B)**. IS, internal standard.

Six carotenoid components were detected in the petals of *O. fragrans* via HPLC-DAD and HPLC-APCI-MS. **Figure 5** shows the separation of these carotenoids in the HPLC chromatogram. The structures of these carotenoids were identified according to the mass spectra and UV-vis absorption spectra data (**Table 3**) (Rivera and Canela-Garayoa, 2012; Van Breemen et al., 2012). Due to the loss of *m/z* 18 u in the fragments, peaks c1, c3 and c4 were identified as hydroxylated carotenoids. Among these three components, peak c1 had a protonated molecule at *m/z* 569, which was identical to lutein and zeaxanthin. This peak was eventually confirmed to be lutein by a corresponding standard. In addition, peak c2 was identified as zeaxanthin by comparison with the retention time and UV-vis absorption spectra of a commercial standard. Peaks c3 and c4 had the same protonated molecules and similar fragmentation patterns. Peak c4 was assigned to β-cryptoxanthin by co-elution with the commercial standard. Additionally, peak c3 was tentatively identified as α-cryptoxanthin according to the literature (Rivera et al., 2014). Although peaks c5 and c6 were protonated at *m/z* 537 and produced the fragment at *m/z* 457 [M + H-80]^+^ (loss of methyl-cyclopentadiene), they were easily distinguished according to the UV-visible spectrum. Additionally, authentic standards were used to confirm the identities of peaks c5 and c6 as α-carotene and β-carotene, respectively.

**Table 3:** HPLC-APCI-MS^2^ analysis and tentative structures of carotenoids in petals of *O. fragrans.*

Peak	tR(min)	λmax (nm)	% (III/II)	(+) APCI-MS^2^ (m/z)	Tentative compound	Reference
c1	15.05	444, 471	63	569.4[M + H]^+^, 551.4[M + H-18]^+^	Lutein	Standard
c2	16.54	450, 476	25	569.4[M + H]^+^	Zeaxanthin	Standard
c3	18.99	440, 470	23	553.4[M + H]^+^, 535.4[M + H-18]^+^, 461.4[M + H-72]^+^	α-Cryptoxanthin	Rivera et al., 2014
c4	19.86	450, 475	33	553.4[M + H]^+^, 535.4[M + H-18]^+^, 461.4[M + H-72]^+^	β-Cryptoxanthin	Standard
c5	21.46	445, 472	62	537.4[M + H]^+^, 457.4[M + H-80]^+^	α-Carotene	Standard
c6	23.18	451, 476	31	537.4[M + H]^+^, 457.4[M + H-80]^+^	β-Carotene	Standard

Corresponding to TCs, the contents of all carotenoid components in the petals of cultivars from the orange-red cluster were also considerably higher than those of the yellowish-white cluster (**Supplementary Table S2**). To ascertain the major carotenoid constituents contributing to *O. fragrans* flower color, a MLR analysis was performed. Because *L^∗^* and *a^∗^* were the main values distinguishing the cultivars and were largely affected by the carotenoids level. These two parameters were selected as the dependent variables, and the proportions of the six carotenoid components among the TCs were set as the independent variables. The MLR results were as follows (*P* < 0.05).

*L^∗^* = 97.184-0.528^∗^α-carotenoid-0.287^∗^β-carotenoid (R^2^= 0.860)

*a^∗^* = -28.336 + 1.103^∗^ α-carotenoid + 0.556^∗^ β-carotenoid (*R*^2^= 0.877)

Therefore, according to the equations based on the MLR analysis, α-carotenoid and β-carotenoid were considered the predominant carotenoid components affecting the flower color of *O. fragrans*, both of which had a negative effect on the value of *L^∗^*, and a positive effect on the value of *a^∗^*.

### Changes in Carotenoid Concentrations During Flowering Development

Since carotenoids were the dominate pigments affecting the flower coloration of *O. fragrans*, changes in carotenoid compositions and contents of the 24 cultivars during the flowering process were further analyzed. Therefore, total carotenoids and the six carotenoid components in the petals at the four flowering stages were used for quantitative analysis by HPLC-DAD (**Supplementary Figures S1–S7**). The concentrations of the six carotenoid components and TCs in the petals of orange-red cultivars (the petals of ‘Yingye Dangui,’ an Aurantiacus Group cultivar with a lighter color, at S1 was an exception) were always higher than those of yellowish-white cultivars during the flowering process. The total carotenoid contents of most cultivars from the Luteus Group (‘Liuye Jingui,’ which has pale yellow flowers was an exception) and Aurantiacus Group tended to increase during flowering development, with the largest concentration observed at S4 (**Supplementary Figure S1**). However, except for ‘Wanyin Gui’ with deeper yellow flowers, the TC levels of the other cultivars from the Albus Group increased initially and then decreased at S4.

**FIGURE 6:**
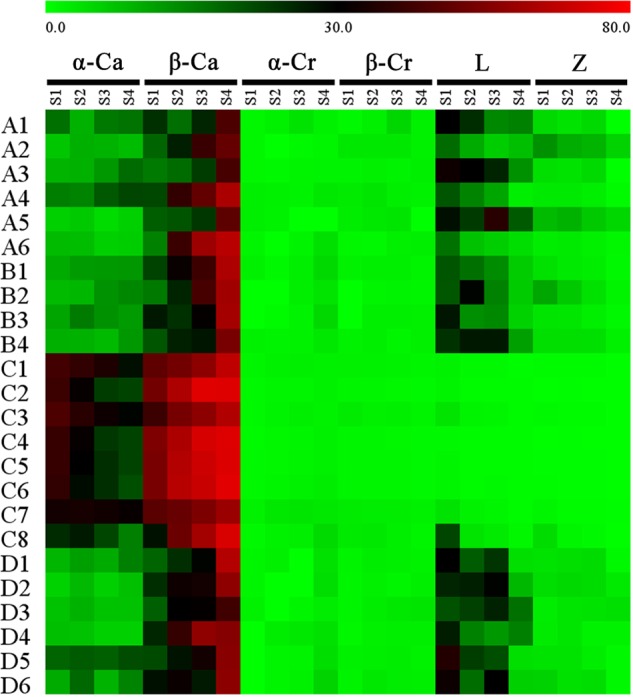
Proportion of each carotenoid component in the total carotenoids of the petals at different flowering stages. Data were expressed as percentage. α-Ca, α-carotene; β-Ca, β-carotene; α-Cr, α-cryptoxanthin; β-Cr, β-cryptoxanthin; L, lutin; Z, zeaxanthin.

Proportional changes in the carotenoid components were also different among the cultivars (**Figure 6**). In the petals of orange-red cultivars, β-carotene and α-carotene were present in the highest amounts among all carotenoid constituents. For most of the yellowish-white cultivars, β-carotene was the predominant component, followed by lutein and α-carotene, e.g., many cultivars contained more lutein than α-carotene. Furthermore, ‘Jiulong Gui’ and ‘Chenghuang Sijigui’ of this cluster contained higher levels of lutein (38.10 and 29.98%) than β-carotene (23.44 and 27.05%) at S3. As the major carotenoid component in *O. fragrans* petals, β-carotene concentrations increased and became greater than α-carotene concentrations during the flowering process in most cultivars, while the relative contents of α-carotene were not consistent in yellowish-white cultivars and obviously decreased in orange-red cultivars. The accumulation patterns of the four xanthophyll constituents, α-cryptoxanthin, β-cryptoxanthin, lutein and zeaxanthin, also differed depending on the cultivars. Lutein, which is the final product of the β, 𝜀-branch, exhibited decreased relative contents in the petals of yellowish-white cultivars and remained in very low proportions in orange-red cultivars, although the concentrations were higher in the latter group (**Supplementary Figure S6**). The content of zeaxanthin, which is the downstream product of the β, β-branch, also tended to decrease during the flowering process. On the other hand, the levels of α-cryptoxanthin and β-cryptoxanthin which are the intermediate products of the β, 𝜀-branch and β, β-branch, respectively, were also significantly different in the two clusters based on flower color. Due to the continuous increase in the α-cryptoxanthin content, the final concentrations of β-cryptoxanthin in the petals of orange-red cultivars were lower than the α-cryptoxanthin concentrations but relatively higher than the lutein concentrations. However, for the yellowish-white cluster, both α-cryptoxanthin and β-cryptoxanthin accumulated at dramatically lower levels than lutein (**Supplementary Figures S4–S6**).

### Relationship Between Gene Expression and Carotenoid Composition

According to our previous research (Zhang et al., 2016), 16 genes involved in the carotenoid metabolism pathway were selected for analysis of their expression levels in the petals of the 24 cultivars at the four flowering stages (**Supplementary Figures S8–S23**). To explore the contributions of gene expression profiles to carotenoid accumulation in *O. fragrans*, a Pearson correlation analysis was performed between relative gene expression levels and total carotenoid contents at the four flowering stages (**Table 4**). Among the carotenoid biosynthetic genes, the expression of *OfZDS1* was positively correlated (*P* < 0.05) with TCs at S2 and S3. *OfCHYE1* expression had a significant correlation only with carotenoid accumulation at S2. For the genes involved in carotenoid degradation, *OfNCED3* expression unexpectedly showed a highly positive correlation (*P* < 0.01) with the total carotenoid concentration. Notably, a significant negative correlation was observed between *OfCCD4-1* expression and carotenoid accumulation throughout the first three flowering stages. In addition, the expression levels of *OfCCD4-1* in cultivars from the yellowish-white cluster were much higher than those in orange-red cultivars (**Supplementary Figure S23**), which showed an inverse correlation with the accumulation patterns of total carotenoids (**Supplementary Figure S1**).

**Table 4:** The Pearson correlation between relative gene expression and total carotenoids content at different flowering stages.

Gene	Different flowering stages			
	S1	S2	S3	S4
*OfPSY1*	–0.162	–0.183	0.041	–0.276
*OfPDS1*	0.197	0.079	0.068	–0.205
*OfZ-ISO1*	–0.134	0.270	0.016	–0.157
*OfZDS1*	–0.012	**0.499^∗^**	**0.486^∗^**	–0.208
*OfCRTISO*	0.055	0.253	0.304	0.044
*OfLCYE1*	–0.028	–0.090	–0.154	–0.163
*OfLCYB1*	–0.155	0.227	–0.051	–0.137
*OfCHYE1*	–0.122	**0.459^∗^**	–0.109	–0.019
*OfCHYB2*	–0.159	–0.345	–0.179	–0.217
*OfZEP1*	–0.151	–0.007	–0.106	–0.125
*OfVDE1*	–0.096	–0.262	–0.175	–0.226
*OfNSY1*	–0.166	0.192	–0.049	–0.142
*OfNCED3*	0.195	0.057	0.087	**0.589^∗∗^**
*OfCCD1-1*	–0.169	–0.139	–0.123	–0.242
*OfCCD1-2*	–0.137	–0.059	–0.125	–0.156
*OfCCD4-1*	**–0.446^∗^**	**–0.534^∗∗^**	**–0.522^∗∗^**	–0.346

*^∗^Correlation is significant at the 0.05 level. ^∗∗^Correlation is significant at the 0.01 level.*

To explain the different proportions of carotenoid components in the two clusters, the expression levels of related genes in two branches were compared. In our research, although the expression patterns of *OfLCYB1* in different cultivars showed no regularity, *OfLCYE1* expression tended to decrease in most cultivars (**Supplementary Figures S13, S14**). Therefore, the expression levels of *OfLCYE1* became much lower than those of *OfLCYB1* during the flowering process, especially at S3 and S4. Additionally, *OfCHYB2* expression was observed to be lower in most cultivars from the orange-red cluster than that in cultivars from the yellowish-white cluster, while the expression levels of *OfCHYE1, OfZEP1*, and *OfVDE1* showed no significant differences between the two clusters.

### Diversity of Carotenoid Composition and Gene Expression in Different Tissues

**FIGURE 7:**
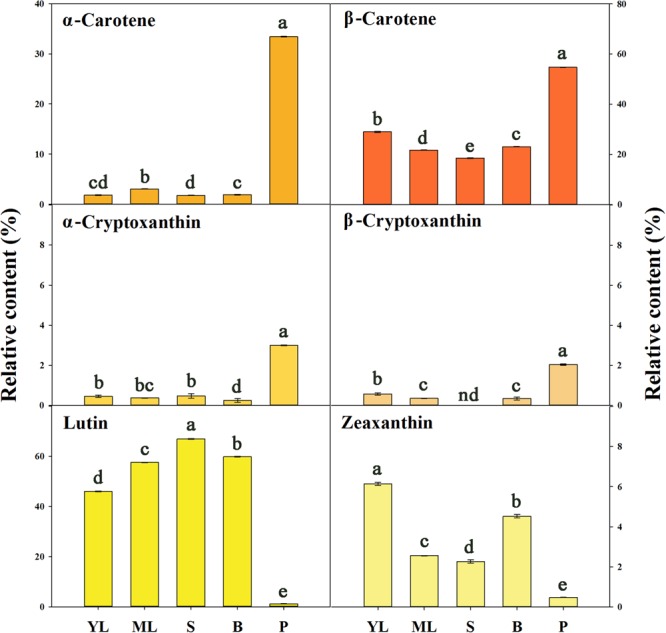
The relative contents of carotenoid components in different tissues of ‘Yanhong Gui.’ YL, young leaves; ML, mature leaves; S, stems; B, flower buds; P, petals. Significant differences (*P* < 0.05 by LSD) are indicated by different letters.

An Aurantiacus cultivar, ‘Yanhong Gui,’ which has abundant carotenoids in its flowers was used to compare the carotenoid compositions in different tissues. According to the HPLC chromatograms (**Supplementary Figure S24**) of carotenoid extracts from young leaves, mature leaves, stems, flower buds and petals, the TC content in the petals was substantially higher than those in the other tissues. Stems and buds contained the lowest levels of TCs. The proportions of each component among the TCs in these tissues were significantly different (**Figure 7**). In the petals, α-carotene (33.41%) and β-carotene (54.73%) were the main constituents, whereas lutein and zeaxanthin only accounted for 1.13 and 0.48% of the TC content, respectively. In contrast, lutein accounted for 45.97 – 66.93% of the TC content in green tissues, representing the most abundant component in these green tissues. Meanwhile, zeaxanthin accounted for 2.27 – 6.14% of the TC content in green tissues, also reflecting a higher proportion than that in petals, which contained higher relative contents of α-cryptoxanthin and β-cryptoxanthin.

**FIGURE 8:**
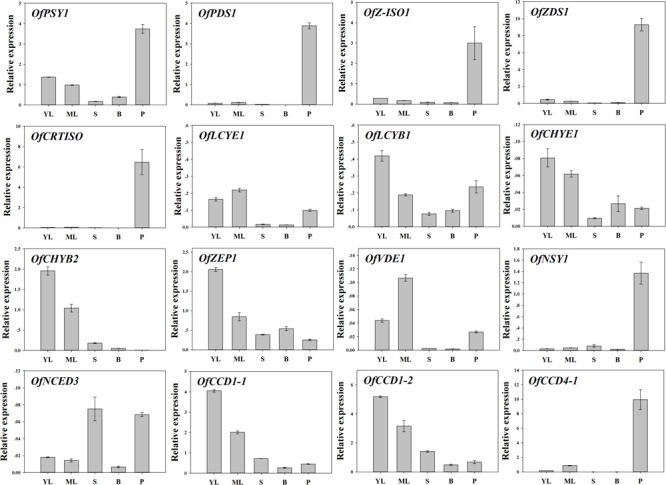
Expression patterns of carotenogenic genes in different tissues of ‘Yanhong Gui.’ YL, young leaves; ML, mature leaves; S, stems; B, flower buds; P, petals.

To examine the regulation of carotenoid accumulation in different tissues, the differential expression levels of carotenoid biosynthetic genes were analyzed (**Figure 8**). Consistent with TC accumulation patterns, *OfPSY1, OfPDS1, OfZ-ISO1, OfZDS1*, and *OfCRTISO* in the upstream pathways were highly expressed in the petals compared with their expression levels in the other tissues. Considering the genes related to the β, 𝜀- and β, β-branches, the expression profiles of *OfLCYB1* in almost all tissues were higher than those of *OfLCYE1*. The expression levels of downstream genes, such as *OfCHYE1, OfCHYB2*, and *OfZEP1*, which are involved in xanthophyll biosynthesis, were much higher in the green tissues. In addition, *OfVDE1* expression in leaves was also higher than that in petals. Regarding the genes of the degradation pathway, the expression levels of *OfCCD1-1* and *OfCCD1-2* were a negatively correlated with the concentration of carotenoids, whereas the expression levels of *OfNCED3* and *OfCCD4-1*, which were highly expressed in petals, showed no correlation with the TC content.

## Discussion

### Pigment Compositions Contributed to the Coloration of Petals

Flower color has been an superficial characteristic used for classification of *O. fragrans* cultivars. Traditionally, the colorific distinctions of three autumn cultivar groups were described based on the range of the RHSCC (Xiang and Liu, 2007). In our study, the flower colors of many cultivars were consistent with their affiliations with cultivar groups, whereas some cultivars belonging to the Albus Group and Luteus Group had unclear classifications. For instance, the cultivars in the Albus Group, such as ‘Yu Linglong’ (an RHSCC value of 9A) showed a yellow flower color, while ‘Wanyin Gui’ (RHSCC value of 13A-13B) had deep yellow flowers. However, the flower colors of ‘Hangzhou Huang’ (RHSCC value of 7A-7B) and ‘Liuye Jingui’ (RHSCC value of 8B-8C) in the Luteus Group were very pale. Combined with the results of the CIE*L^∗^a^∗^b^∗^* system, all cultivars in the Aurantiacus Group, which exhibited a reddish color, were defined as one cluster, whereas the other three groups were classified together and can not be distinguished due to the similarity of their lighter colors. Some controversy exists in the classification of some cultivars in the Albus Group and Luteus Group, and some modification should probably be applied for further cultivar distinction. Therefore, follow-up research focused on the differences between the two major clusters.

Red flower can be produced by high concentrations of carotenoids, anthocyanins, or a combination of both pigments (Tanaka et al., 2008; Yuan et al., 2013; Ng and Smith, 2016a,b). According to our investigation, no chromatographic peak under 520 nm was detected, indicating that no anthocyanins were present in the tested cultivars, even in the cultivars with reddest flowers in the Aurantiacus Group. The absence of anthocyanins is consistent with previous work by Han et al. (2015), who found that *OfANS* was not detected in either ‘Dangui’ (Aurantiacus) or ‘Yingui’ (Albus). Abundant flavonoids were detected in the petals, but little difference was observed in the quantity of flavonoids among the cultivar groups. Significant correlations between carotenoid concentrations and parameters of color phenotypes were observed not only in cultivars from different color groups but also in cultivars within the same color group, leading to the conclusion that carotenoids play a predominant role in producing distinct colors in different *O. fragrans* cultivars, which is consistent with previous research (Han et al., 2013, 2014). Furthermore, the coloration effects of flavonoid components, such as rutin, isoquercitrin, quercitrin, and quercitrin reported in the petals of *O. fragrans* (Hung et al., 2012; Chen et al., 2015), were much weaker than those of carotenoids producing colors ranging from vivid yellow to bright red. Therefore, in some cultivars of the Albus Group or the Luteus Group, flavonoids can confer their very pale-yellow color only when the carotenoid concentration is extremely low (Tanaka et al., 2008). Taken together, the difference in the flower coloration of *O. fragrans* are mainly attributable to the level of carotenoids, and the color of petals becomes more reddish and deeper with the accumulation of carotenoids. Flavonoids were speculated to provide only background color in *O. fragrans* flowers.

### The Carotenogenic Pathway in Petals of Different Cultivars

**FIGURE 9:**
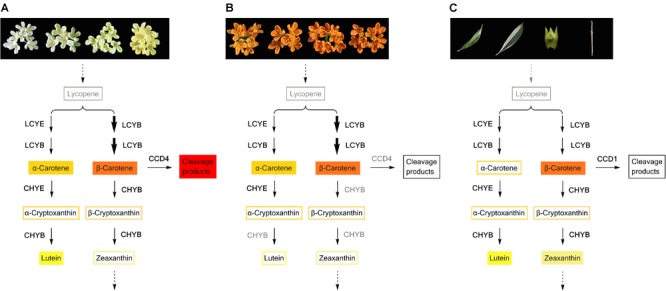
Carotenoid biosynthesis flux in petals of yellowish-white cultivars **(A)** and orange-red cultivars **(B)**, and green tissues of *O. fragrans* **(C)**. The carotenoid components with filled color denote the predominant constituents. Decreased *OfLCYE* expression during flowering time, led to a higher concentration of β, β-branch products in both yellowish-white cultivars and orange-red cultivars, which were indicated by bolder arrows. In graph **(B)**, the enzymes such as CHYB and CCD4 in gray color indicate that the expression levels of the corresponding genes were down-regulated in orange-red cultivars compared with those in yellowish-white cultivars.

Many plant species can accumulate special carotenoid constituents in their flowers, such as astaxanthin in the blood-red flowers of *Adonis annua* and *Adonis aestivalis* (Seybold and Goodwin, 1959; Cunningham and Gantt, 2011), capsanthin in orange and red tepals of lily (Jeknić et al., 2012), and lutein epoxides in and yellow petals of chrysanthemum (Kishimoto et al., 2004). Previous research has reported small amounts of β-carotene in the petals of ‘Zi Yingui’ (an Albus Group cultivar), high concentrations of lutein and β-carotene as well as low levels of α-carotene in the petals of ‘Jingui’ (a Luteus Group cultivar), and abundant α-carotene and β-carotene in the petals of ‘Chenghong Dangui’ (an Aurantiacus Group cultivar), representing the main carotenoid components in *O. fragrans* flowers (Han et al., 2014). In our present study, six carotenoid components were detected in the petals of 24 *O. fragrans* cultivars, providing specific information on metabolic products to study the flux of carotenoid biosynthesis in the two groups of cultivars (**Figures 9A,B**). Without considering the differences in total carotenoid concentrations between the cultivar groups, we found that the petals of yellowish-white cultivars exhibited high contents of β-carotene, lutein and α-carotene (in order from higher to lower levels), while the petals of orange-red cultivars contained much higher levels of β-carotene and α-carotene than any other constituents. Additionally, the β-carotene content in the β, β-branch increased during flowering and ultimately showed the highest content in most cultivars. According to many reports, the balance of products in the β, 𝜀-branch and β, β-branch was affected by the activities of LCYB and LCYE, which determine the carotenoid composition in many plant species (Moehs et al., 2001; Kishimoto and Ohmiya, 2006; Chiou et al., 2010; Yamamizo et al., 2010). In the petals of *O. fragrans*, higher levels of *OfLCYE* were found in ‘Jingui’ than in ‘Zi Yingui’ and ‘Chenghong Dangui’ (Han et al., 2014). Considering the populations consisting of many cultivars, the expression of *OfLCYB1* as well as *OfLCYE1* showed no significant differences between the two clusters in our research. However, the distribution of the carotenoid flux was due to decreased levels of *OfLCYE1*. representing a limitation for β, 𝜀-carotenoid production and accounting for the decreased concentrations of α-carotene in orange-red cultivars of *O. fragrans*, and reduced lutein levels in yellowish-white cultivars. Comparison of the different *O. fragrans* groups revealed no obvious differences in the transcriptional levels of downstream genes of xanthophyll biosynthesis such as *CHYE, ZEP, VDE* and *NSY*. However, down-regulation of *OfCHYB2* in orange-red cultivars led to the accumulation of upstream products. In contrast, the higher proportions of lutein in yellowish-white cultivars were attributable to relatively higher *OfCHYB2* expression. Similarly, *CHYB* played an important role in the diverse carotenoid compositions of yellow and orange *Oncidium* cultivars (Chiou et al., 2010). As reported in tomato and *Ipomoea* plants, CHYB is a key enzyme responsible for carotenoid concentrations in white petals and yellow petals (Galpaz et al., 2006; Yamamizo et al., 2010). Therefore, differential expression of *OfCHYB2* was speculated to be one of factors impacting the total carotenoid levels in *O. fragrans*.

The total carotenoid concentrations showed notable diversity between the two color groups of *O. fragrans*. The transcript abundance of only a few synthetic genes and degradation genes was correlated with the accumulation patterns of total carotenoids at different flowering stages. Among the upstream genes, *OfZDS1* expression was positively correlated (*P* < 0.05) with total carotenoids at two flowering stages (**Table 4**), while in the downstream pathway, *OfCHYE1* levels showed positive correlations with total carotenoids at the half opening stage. *ZDS* was reported to be involved in the high lycopene concentration in carrot root of red cultivars (Clotault et al., 2008). The function of *CHYE* was confirmed in both an *Arabidopsis lut5* mutant and orange-rooted carrots with defective *CYP97A3*, which caused accumulation of α-carotene (Kim and DellaPenna, 2006; Arango et al., 2014). NCEDs are involved in the degradation of carotenoids and the production of ABA, which participates in the senescence of flowers (Trivellini et al., 2011; Lü et al., 2014). At the last flowering stage, the positive correlation (*P* < 0.01) between the transcriptional levels of *OfNCED3* and the total carotenoid contents did not seem to be the most important factor for carotenoid accumulation; the most crucial factor determining the diversity of carotenoid concentrations was the differential expression level of *OfCCD4-1*, which showed a significant correlation with the total carotenoid content throughout nearly all flowering process. Similar to ‘Zi Yingui’ (Han et al., 2014), up-regulated levels of *OfCCD4-1* in the petals of yellowish-white cultivars in this study, led to considerably lower concentrations of each component compared with those in orange-red cultivars. Reportedly, *CCD4* also has a significant effect on carotenoid accumulation in chrysanthemum petals, potato tubers, and yellow-fleshed and white-fleshed peaches (Ohmiya et al., 2006; Campbell et al., 2010; Brandi et al., 2011; Rodrigo et al., 2013). The enzymatic activities of CCD1 and CCD4 are responsible for the cleavage of double bonds in multiple carotenoid substrates at different sites and for the manifestation of some flavors and aromas (Huang et al., 2009; Baldermann et al., 2010). As shown in **Figure 9A**, higher levels of cleavage products, such as α-ionone and β-ionone released from flowers of cultivars in the Albus Group, Luteus Group and Asiaticus Group (Xin et al., 2013; Cai et al., 2014), were consistent with the expression patterns of *OfCCD4-1*. However, two transcripts of *OfCCD1* were expressed in different cultivars, without an obvious relation with the carotenoid concentration, even though OfCCD1 has powerful activity against α-carotene and β-carotene (Baldermann et al., 2010), probably because CCD1 enzymes are located in the cytosol while carotenoids in petals mainly accumulate in chromoplasts, thus providing limited substrates for catalysis (Frusciante et al., 2014).

### The Carotenogenic Pathway in Different Tissues

Carotenoids can be classified as ‘chloroplast-type carotenoids’ and ‘chromoplast-type carotenoids,’ based on their locations in different types of plastids (Yamamizo et al., 2010). These carotenoids have distinct functions; chloroplast-type carotenoids are mainly produced in photosynthetic tissues such as leaves and are essential pigments involved in photosynthesis, while chromoplast-type carotenoids which mainly accumulate in flowers and fruits, conferring yellow, orange and red colors to attract insects and animals for pollination or seed dispersal (Cazzonelli and Pogson, 2010; Yuan et al., 2015). In our study, while large amounts of β-carotene and α-carotene accumulated in the flowers of ‘Yan Honggui,’ lutein followed by β-carotene and zeaxanthin were the main carotenoid components of leaves, stems and flower buds. As shown in **Figure 9C**, downstream genes such as *OfCHYE1, OfCHYB2, OfZEP1*, and *OfVDE1* showed higher expression levels in green tissues, especially in leaves. Among carotenoids, since lutein, β-carotene, violaxanthin, neoxanthin and zeaxanthin are typically stored in leaves (Cazzonelli and Pogson, 2010), lutein and zeaxanthin have been suggested to be the most effective xanthophylls participating in photoprotection by quenching excited intermediates such as triplet chlorophylls and singlet oxygen molecules (Dall’Osto et al., 2010; Domonkos et al., 2013). Therefore, the carotenoid composition in the green tissues of *O. fragrans* can exert a protective function. Additionally, chromoplast-type carotenoids are mostly present in the esterified form for the sequestration of carotenoids into chromoplasts, whereas the chloroplast-type carotenoids which are primarily present in free form (Yamamizo et al., 2010), can likely be catalyzed due to high *OfCCD1* expression in green tissues. This degradation may be involved in the final carotenoid concentrations.

Compared with green tissues, the abundant carotenoids in the petals of ‘Yan Honggui’ as well as other Aurantiacus Group cultivars were attributable not only to lower expressions levels of downstream genes and degradation genes but also to up-regulated expression levels of genes in the upstream pathway, including *OfPSY1, OfPDS1, OfZ-ISO1, OfZDS1*, and *OfCRTISO*. Notably, the combination of a high lutein percentage in flower buds, higher proportions of α-carotene, β-carotene, α-cryptoxanthin and β-cryptoxanthin in the petals of Aurantiacus Group cultivars, as well as decreased lutein levels in yellowish-white cultivars, indicated that a shift from ‘chloroplast-type carotenoids’ to ‘chromoplast-type carotenoids’ occurs during the flowering process.

### Transcriptional Regulation of Carotenogenic Genes and the Assumed Phylogeny of *O. fragrans* Cultivars

In summary, the present study suggested that different carotenoid compositions among *O. fragrans* cultivars were mainly due to differential expression levels of downstream genes such as *OfLCYE1* and *OfCHYB2*. Additionally, profound diversity in total carotenoid concentrations was identified by the transcript levels of *OfCCD4-1*. Considering the specificity of carotenoid biosynthesis in different tissues of *O. fragrans*, determination of the carotenoid compositions and concentrations depended on almost all the genes related to the corresponding pathways (**Figure 9**). Therefore, transcriptional regulation of carotenogenic genes plays an important role in carotenoid production in *O. fragrans*. The transcript levels of many carotenoid-related genes could be regulated by multiple factors. Reportedly, the promoters of *GlPDS, GlZDS, GlLYCB, GlLYCE, GlBCH*, and *GlZEP* isolated from *Gentiana lutea* contained *cis*-regulatory motifs responding to methyl jasmonate (CGTCA) and ethylene (ATCTA) (Zhu et al., 2014). In addition to these two phytohormones, auxin and ABA affect carotenoid accumulation in tomato fruits directly or by regulating fruit ripening related transcription factors (Liu et al., 2012, 2015). The carotenogenic gene promoters also show tissues-specific activities, leading to the regulation of these genes during developmental changes from non-chromoplast-containing tissues to chromoplast-containing tissues (Dalal et al., 2010; Yang et al., 2012; Zhu et al., 2014). In addition, transcription factors may participate in regulating the structure genes involved in carotenoid metabolism, which have been reported in some plants. For example, RCP1, an R2R3-MYB identified in *Mimulus lewisii* flowers, can positively regulate the entire carotenoid biosynthesis pathway (Sagawa et al., 2016). In *Citrus* plants, CrMYB68 (an R2R3-MYB transcriptional factor) and CsMADS6 (an MADS transcription factor) are involved in carotenoid metabolism by directly binding to the promoters of carotenogenic genes (Zhu et al., 2017; Lu et al., 2018). *OfWRKY3* isolated from the petals of *O. fragrans* is a positive regulator of *OfCCD4* (Han et al., 2016). However, information of transcription factors in *O. fragrans* is relatively limited. Therefore, to elucidate the mechanism generating the in transcriptional diversity of carotenogenic genes, more transcription factors must be isolated in *O. fragrans*. Carotenoid metabolism involves multiple influencing factors and is a complex process. In addition to the mechanisms described above, carotenoid accumulation in *O. fragrans* may also be controlled by a feedback effect, enzyme activity, sink capacity and epigenetic inheritance (Cazzonelli and Pogson, 2010; Zhu et al., 2010; Yuan et al., 2015), which should to be investigated in the future.

The variations in carotenoid metabolism in different *O. fragrans* cultivars are speculated to be closely related to cultivars’ genetic backgrounds. Genetic relationships among different cultivar clusters have been investigated using molecular markers, but no consistent conclusions have been reached. For example, using amplified fragment length polymorphism (AFLP) markers, the closest genetic relationship was suggested to exist between the Luteus group and the Aurantiacus group, and the Asiaticus group had a distant relationship with the other three autumn groups (Yuan et al., 2011). However, when microsatellite markers were used to classify *O. fragrans* cultivars, the result revealed that cultivars in the Asiaticus and Albus groups, which are less differentiated from wild *O. fragrans*, were genetically mixed, whereas cultivars in the Luteus and Aurantiacus groups were partially genetically differentiated (Duan et al., 2013). In our study, many cultivars in the Albus Group, Luteus Group, and Asiaticus Group showed high similarity based on phytochemistry analysis as well as transcriptional levels of related genes. Many *O. fragrans* cultivars originated from wild *O. fragrans* through crossbreeding as well as mutation (Xiang and Liu, 2007). Our previous study found that a mutant with phenotypes of the Aurantiacus group contained extremely higher concentrations of α-carotene, β-carotene as well as total carotenoids in the petals compared to those in the stock plant, a cultivar from the Luteus group (Wang et al., 2017). In the petals of *Mimulus lewisii*, the presence and absence of carotenoids is controlled by a single QTL at the *YUP* locus (Bradshaw and Schemske, 2003). In other plants such as cucumber, an SNP codes for an amino acid change in a gene encoding a putative β-carotene hydroxylase, leading to extensive accumulation of β-carotene in wild cucumber fruit from the Xishuangbanna group, while the cultivated populations usually contain limited carotenoid contents (Qi et al., 2013). Since the *Y* locus regulates carotenoid accumulation in carrot taproot, both yellow and dark orange carrots rather than white carrots have a frameshift mutation in DCAR_032551, which contains a 212-nt insertion in its second exon (Iorizzo et al., 2016). In addition, insertion of transposable elements in *CCD4* of *Brassica* species (Zhang et al., 2015) or loss of *CmCCD4a* in the chrysanthemum genome (Ohmiya et al., 2006) are also responsible for generating yellow petals by interfering with carotenoid degradation. Consequently, we can speculate that variations in some genes may have occurred during plant domestication, thus affecting the formation of cultivars based on specific phenotypes. However, this speculation requires investigation in further research.

## Conclusion

In this research, selected *O. fragrans* cultivars of four groups were divided into two clusters: an orange-red cluster and a yellowish-white cluster. Carotenogenic pathways in both clusters tended to generate β, β-branched products due to down-regulated *OfLCYE1*. In the orange-red cluster, low expression levels of downstream genes and carotenoid cleavage gene such as *OfCCD4* caused extensive accumulation of carotenoids (especially α-carotenoid and β-carotenoid), resulting in a reddish flower color. In the yellowish-white cluster, high expression levels of downstream genes such as *OfCHYB2* resulted in conversion of more α-carotenoid into lutein, and up-regulated *OfCCD4* reduced the total carotenoid content, which led to the formation of a pale petal color (white or yellow). Compared with green photosynthetic tissues, up-regulated genes in the upstream pathway as well as down-regulated downstream genes caused tissue-specific accumulation of chromoplast-type carotenoids in the orange-red petals of *O. fragrans*. This work revealed the flower coloration patterns of different *O. fragrans* cultivars, which were affected by distinct carotenoid metabolism processes.

## Author Contributions

HZ and CZ designed the research. YW and CZ performed the experiments. YW, BD, and JF analyzed the data and results. YW wrote the paper. HZ, CZ, BD, JF, and SH revised the paper.

## Conflict of Interest Statement

The authors declare that the research was conducted in the absence of any commercial or financial relationships that could be construed as a potential conflict of interest.

## References

[B1] Arango J. Jourdan M. Geoffriau E. Beyer P. Welsch R. (2014). Carotene hydroxylase activity determines the levels of both α-carotene and total carotenoids in orange carrots. *Plant Cell* 26 2223–2233. 10.1105/tpc.113.122127 24858934 PMC4079379

[B2] Asensi-Fabado M. A. Munné-Bosch S. (2010). Vitamins in plants: occurrence, biosynthesis and antioxidant function. *Trends Plant Sci.* 15 582–592. 10.1016/j.tplants.2010.07.003 20729129

[B3] Baldermann S. Kato M. Kurosawa M. Kurobayashi Y. Fujita A. Fleischmann P. (2010). Functional characterization of a carotenoid cleavage dioxygenase 1 and its relation to the carotenoid accumulation and volatile emission during the floral development of *Osmanthus fragrans* Lour. *J. Exp. Bot.* 61 2967–2977. 10.1093/jxb/erq123 20478967

[B4] Bradshaw H. D. Schemske D. W. (2003). Allele substitution at a flower colour locus produces a pollinator shift in monkeyflowers. *Nature* 426 176–178. 10.1038/nature02106 14614505

[B5] Brandi F. Bar E. Mourgues F. Horváth G. Turcsi E. Giuliano G. (2011). Study of ’Redhaven’ peach and its white-fleshed mutant suggests a key role of CCD4 carotenoid dioxygenase in carotenoid and norisoprenoid volatile metabolism. *BMC Plant Biol.* 11:24. 10.1186/1471-2229-11-24 21269483 PMC3045293

[B6] Cai X. Mai R. Z. Zou J. J. Zhang H. Y. Zeng X. L. Zheng R. R. (2014). Analysis of aroma-active compounds in three sweet osmanthus (*Osmanthus fragrans*) cultivars by GC-olfactometry and GC-MS. *J. Zhejiang Univ. Sci. B* 15 638–648. 10.1631/jzus.B1400058 25001223 PMC4097373

[B7] Campbell R. Ducreux L. J. M. Morris W. L. Morris J. A. Suttle J. C. Ramsay G. (2010). The metabolic and developmental roles of carotenoid cleavage dioxygenase4 from potato. *Plant Physiol.* 154 656–664. 10.1104/pp.110.158733 20688977 PMC2949026

[B8] Cazzonelli C. I. (2014). Carotenoids in nature: insights from plants and beyond. *Funct. Plant Biol.* 38 833–847. 10.1071/FP11192 32480941

[B9] Cazzonelli C. I. Pogson B. J. (2010). Source to sink: regulation of carotenoid biosynthesis in plants. *Trends Plant Sci.* 15 266–274. 10.1016/j.tplants.2010.02.003 20303820

[B10] Chen G.-L. Chen S.-G. Xie Y.-Q. Chen F. Zhao Y.-Y. Luo C.-X. (2015). Total phenolic, flavonoid and antioxidant activity of 23 edible flowers subjected to in vitro digestion. *J. Funct. Foods* 17 243–259. 10.1016/j.jff.2015.05.028

[B11] Chiou C.-Y. Pan H.-A. Chuang Y.-N. Yeh K.-W. (2010). Differential expression of carotenoid-related genes determines diversified carotenoid coloration in floral tissues of *Oncidium* cultivars. *Planta* 232 937–948. 10.1007/s00425-010-1222-x 20635095

[B12] Clotault J. Peltier D. Berruyer R. Thomas M. Briard M. Geoffriau E. (2008). Expression of carotenoid biosynthesis genes during carrot root development. *J. Exp. Bot.* 59 3563–3573. 10.1093/jxb/ern210 18757491

[B13] Cunningham F. X. Gantt E. (2011). Elucidation of the pathway to astaxanthin in the flowers of *Adonis aestivalis*. *Plant Cell* 23 3055–3069. 10.1105/tpc.111.086827 21862704 PMC3180810

[B14] Dalal M. Chinnusamy V. Bansal K. C. (2010). Isolation and functional characterization of *Lycopene β-cyclase (CYC-B)* promoter from *Solanum habrochaites*. *BMC Plant Biol.* 10:61. 10.1186/1471-2229-10-61 20380705 PMC2923535

[B15] Dall’Osto L. Cazzaniga S. Havaux M. Bassi R. (2010). Enhanced photoprotection by protein-bound vs free xanthophyll pools: a comparative analysis of chlorophyll b and xanthophyll biosynthesis mutants. *Mol. Plant* 3 576–593. 10.1093/mp/ssp117 20100799

[B16] Delpino-Rius A. Eras J. Marsol-Vall A. Vilaró F. Balcells M. Canela-Garayoa R. (2014). Ultra performance liquid chromatography analysis to study the changes in the carotenoid profile of commercial monovarietal fruit juices. *J. Chromatogr. A* 1331 90–99. 10.1016/j.chroma.2014.01.044 24503121

[B17] Domonkos I. Kis M. Gombos Z. Ughy B. (2013). Carotenoids, versatile components of oxygenic photosynthesis. *Prog. Lipid Res.* 52 539–561. 10.1016/j.plipres.2013.07.001 23896007

[B18] Duan Y. Wang X. Xiang Q. Liang L. Li X. Liu Y. (2013). Genetic diversity of androdioecious *Osmanthus fragrans* (Oleaceae) cultivars using microsatellite markers. *Appl. Plant Sci.* 1:1200092. 10.3732/apps.1200092 25202550 PMC4105023

[B19] Frusciante S. Diretto G. Bruno M. Ferrante P. Pietrella M. Prado-Cabrero A. (2014). Novel carotenoid cleavage dioxygenase catalyzes the first dedicated step in saffron crocin biosynthesis. *Proc. Natl. Acad. Sci. U.S.A.* 111 12246–12251. 10.1073/pnas.1404629111 25097262 PMC4143034

[B20] Galpaz N. Ronen G. Khalfa Z. Zamir D. Hirschberg J. (2006). A chromoplast-specific carotenoid biosynthesis pathway is revealed by cloning of the tomato white-flower locus. *Plant Cell* 18 1947–1960. 10.1105/tpc.105.039966 16816137 PMC1533990

[B21] Gonnet J. F. (1998). Colour effects of co-pigmentation of anthocyanins revisited—1. A colorimetric definition using the CIELAB scale. *Food Chem.* 63 409–415. 10.1016/S0308-8146(98)00053-3

[B22] Grotewold E. (2006). The genetics and biochemistry of floral pigments. *Annu. Rev. Plant Bio.* 57 761–780. 10.1146/annurev.arplant.57.032905.105248 16669781

[B23] Han Y. Chen W. Yang F. Wang X. Dong M. Zhou P. (2015). cDNA-AFLP analysis on 2 *Osmanthus fragrans* cultivars with different flower color and molecular characteristics of OfMYB1 gene. *Trees* 29 931–940. 10.1007/s00468-015-1175-6

[B24] Han Y. Li L. Dong M. Yuan W. Shang F. (2013). cDNA cloning of the phytoene synthase (PSY) and expression analysis of PSY and carotenoid cleavage dioxygenase genes in *Osmanthus fragrans*. *Biologia* 68 258–263. 10.2478/s11756-013-0002-z

[B25] Han Y. Wang X. Chen W. Dong M. Yuan W. Liu X. (2014). Differential expression of carotenoid-related genes determines diversified carotenoid coloration in flower petal of *Osmanthus fragrans*. *Tree Genet. Genomes* 10 329–338. 10.1007/s11295-013-0687-8

[B26] Han Y. Wu M. Cao L. Yuan W. Dong M. Wang X. (2016). Characterization of OfWRKY3, a transcription factor that positively regulates the carotenoid cleavage dioxygenase gene OfCCD4 in *Osmanthus fragrans*. *Plant Mol. Biol.* 91 485–496. 10.1007/s11103-016-0483-6 27106478

[B27] Huang F.-C. Molnár P. Schwab W. (2009). Cloning and functional characterization of carotenoid cleavage dioxygenase 4 genes. *J. Exp. Bot.* 60 3011–3022. 10.1093/jxb/erp137 19436048 PMC2718213

[B28] Hung C.-Y. Tsai Y.-C. Li K.-Y. (2012). Phenolic antioxidants isolated from the flowers of *Osmanthus fragrans*. *Molecules* 17 10724–10737. 10.3390/molecules170910724 22960867 PMC6268160

[B29] Iorizzo M. Ellison S. Senalik D. Zeng P. Satapoomin P. Huang J. (2016). A high-quality carrot genome assembly provides new insights into carotenoid accumulation and asterid genome evolution. *Nat. Genet.* 48 657–666. 10.1038/ng.3565 27158781

[B30] Jeknić Z. Morré J. T. Jeknić S. Jevremović S. Subotić A. Chen T. H. H. (2012). Cloning and functional characterization of a gene for capsanthin-capsorubin synthase from *Tiger lily* (*Lilium lancifolium* Thunb. ‘Splendens’). *Plant Cell Physiol.* 53 1899–1912. 10.1093/pcp/pcs128 23008421 PMC3494009

[B31] Kim J. DellaPenna D. (2006). Defining the primary route for lutein synthesis in plants: the role of *Arabidopsis* carotenoid β-ring hydroxylase CYP97A3. *Proc. Natl. Acad. Sci. U.S.A.* 103 3474–3479. 10.1073/pnas.0511207103 16492736 PMC1413914

[B32] Kishimoto S. Maoka T. Nakayama M. Ohmiya A. (2004). Carotenoid composition in petals of chrysanthemum (*Dendranthema grandiflorum* (Ramat.) Kitamura). *Phytochemistry* 65 2781–2787. 10.1016/j.phytochem.2004.08.038 15474564

[B33] Kishimoto S. Ohmiya A. (2006). Regulation of carotenoid biosynthesis in petals and leaves of chrysanthemum (*Chrysanthemum morifolium*). *Physiol. Plant.* 128 436–447. 10.1111/j.1399-3054.2006.00761.x 23643306

[B34] Liu L. Shao Z. Zhang M. Wang Q. (2015). Regulation of carotenoid metabolism in tomato. *Mol. Plant* 8 28–39. 10.1093/mp/ssu121 25578270

[B35] Liu L. Wei J. Zhang M. Zhang L. Li C. Wang Q. (2012). Ethylene independent induction of lycopene biosynthesis in tomato fruits by jasmonates. *J. Exp. Bot.* 63 5751–5761. 10.1093/jxb/ers224 22945939 PMC3467294

[B36] Lü P. Zhang C. Liu J. Liu X. Jiang G. Jiang X. (2014). RhHB1 mediates the antagonism of gibberellins to ABA and ethylene during rose (*Rosa hybrida*) petal senescence. *Plant J.* 78 578–590. 10.1111/tpj.12494 24589134

[B37] Lu S. Zhang Y. Zhu K. Yang W. Ye J. Chai L. (2018). The citrus transcription factor CsMADS6 modulates carotenoid metabolism by directly regulating carotenogenic genes. *Plant Physiol.* 176 2657–2676. 10.1104/pp.17.01830 29463773 PMC5884614

[B38] Ma G. Zhang L. Matsuta A. Matsutani K. Yamawaki K. Yahata M. (2013). Enzymatic formation of β-citraurin from β-cryptoxanthin and zeaxanthin by carotenoid cleavage dioxygenase 4 in the flavedo of citrus fruit. *Plant Physiol.* 163 682–695. 10.1104/pp.113.223297 23966550 PMC3793050

[B39] Moehs C. P. Tian L. Osteryoung K. W. DellaPenna D. (2001). Analysis of carotenoid biosynthetic gene expression during marigold petal development. *Plant Mol. Biol.* 45 281–293. 10.1023/A:1006417009203 11292074

[B40] Mu H. N. Li H. G. Wang L. G. Yang X. L. Sun T. Z. Xu C. (2014). Transcriptome sequencing and analysis of sweet osmanthus (*Osmanthus fragrans* Lour.). *Genes Genomics* 36 777–788. 10.1007/s13258-014-0212-y

[B41] Ng J. Smith S. D. (2016a). How to make a red flower: the combinatorial effect of pigments. *AoB Plants* 8:plw013. 10.1093/aobpla/plw013 26933150 PMC4804202

[B42] Ng J. Smith S. D. (2016b). Widespread flower color convergence in Solanaceae via alternate biochemical pathways. *New Phytol.* 209 407–417. 10.1111/nph.13576 26224118

[B43] Nisar N. Li L. Lu S. Khin N. C. Pogson B. J. (2015). Carotenoid metabolism in plants. *Mol. Plant* 8 68–82. 10.1016/j.molp.2014.12.007 25578273

[B44] Ohmiya A. (2013). Qualitative and quantitative control of carotenoid accumulation in flower petals. *Sci. Hortic.* 163 10–19. 10.1016/j.scienta.2013.06.018

[B45] Ohmiya A. Kishimoto S. Aida R. Yoshioka S. Sumitomo K. (2006). Carotenoid cleavage dioxygenase (CmCCD4a) contributes to white color formation in chrysanthemum petals. *Plant Physiol.* 142 1193–1201. 10.1104/pp.106.087130 16980560 PMC1630759

[B46] Qi J. Liu X. Shen D. Miao H. Xie B. Li X. (2013). A genomic variation map provides insights into the genetic basis of cucumber domestication and diversity. *Nat. Genet.* 45 1510–1515. 10.1038/ng.2801 24141363

[B47] Rivera S. M. Canela-Garayoa R. (2012). Analytical tools for the analysis of carotenoids in diverse materials. *J. Chromatogr. A* 1224 1–10. 10.1016/j.chroma.2011.12.025 22226560

[B48] Rivera S. M. Christou P. Canela-Garayoa R. (2014). Identification of carotenoids using mass spectrometry. *Mass Spectrom. Rev.* 33 353–372. 10.1002/mas.21390 24178708

[B49] Rodrigo M. J. Alquézar B. Alós E. Medina V. Carmona L. Bruno M. (2013). A novel carotenoid cleavage activity involved in the biosynthesis of Citrus fruit-specific apocarotenoid pigments. *J. Exp. Bot.* 64 4461–4478. 10.1093/jxb/ert260 24006419 PMC3808326

[B50] Rodríguez-Concepción M. (2010). Supply of precursors for carotenoid biosynthesis in plants. *Arch. Biochem. Biophys.* 504 118–122. 10.1016/j.abb.2010.06.016 20561506

[B51] Sagawa J. M. Stanley L. E. Lafountain A. M. Frank H. A. Liu C. Yuan Y. W. (2016). An R2R3-MYB transcription factor regulates carotenoid pigmentation in *Mimulus lewisii* flowers. *New Phytol.* 209 1049–1057. 10.1111/nph.13647 26377817

[B52] Schmittgen T. D. Livak K. J. (2008). Analyzing real-time PCR data by the comparative C(T) method. *Nat. Protoc.* 3 1101–1108. 10.1038/nprot.2008.73 18546601

[B53] Seto Y. Sado A. Asami K. Hanada A. Umehara M. Akiyama K. (2014). Carlactone is an endogenous biosynthetic precursor for strigolactones. *Proc. Natl. Acad. Sci. U.S.A.* 111 1640–1645. 10.1073/pnas.1314805111 24434551 PMC3910621

[B54] Seybold A. Goodwin T. W. (1959). Occurrence of astaxanthin in the flower petals of *Adonis annua* L. *Nature* 184(Suppl. 22) 1714–1715. 10.1038/1841714a0 14445279

[B55] Tan B.-C. Joseph L. M. Deng W.-T. Liu L. Li Q.-B. Cline K. (2003). Molecular characterization of the Arabidopsis 9-cis-epoxycarotenoid dioxygenase gene family. *Plant J.* 35 44–56. 10.1046/j.1365-313X.2003.01786.x 12834401

[B56] Tanaka Y. Sasaki N. Ohmiya A. (2008). Biosynthesis of plant pigments: anthocyanins, betalains and carotenoids. *Plant J.* 54 733–749. 10.1111/j.1365-313X.2008.03447.x 18476875

[B57] Trivellini A. Ferrante A. Vernieri P. Serra G. (2011). Effects of abscisic acid on ethylene biosynthesis and perception in *Hibiscus rosa-sinensis* L. flower development. *J. Exp. Bot.* 62 5437–5452. 10.1093/jxb/err218 21841180 PMC3223042

[B58] Tsai T.-H. Tsai T.-H. Chien Y.-C. Lee C.-W. Tsai P.-J. (2008). *In vitro* antimicrobial activities against cariogenic streptococci and their antioxidant capacities: a comparative study of green tea versus different herbs. *Food Chem.* 110 859–864. 10.1016/j.foodchem.2008.02.085 26047271

[B59] Van Breemen R. B. Dong L. Pajkovic N. D. (2012). Atmospheric pressure chemical ionization tandem mass spectrometry of carotenoids. *Int. J. Mass Spectrom.* 312 163–172. 10.1016/j.ijms.2011.07.030 22408388 PMC3293484

[B60] Wang Y. Luo Y. Zhang C. Fu J. Hu S. Zhao H. (2017). Flower color and pigment composition in the petals of bud mutation and its stock plant of *Osmanthus fragrans* ’Jingui’. *Acta Hortic. Sin.* 44 528–536. 10.16420/j.issn.0513-353x.2016-0618

[B61] Xiang Q. Liu Y. (2007). *An Illustrated Monograph of the Sweet Osmanthus Variety in China.* Hangzhou: Zhejiang Science and Technology Press.

[B62] Xin H. Wu B. Zhang H. Wang C. Li J. Yang B. (2013). Characterization of volatile compounds in flowers from four groups of sweet osmanthus (*Osmanthus fragrans*) cultivars. *Can. J. Plant Sci.* 93 923–931. 10.4141/cjps2012-333

[B63] Yamamizo C. Kishimoto S. Ohmiya A. (2010). Carotenoid composition and carotenogenic gene expression during *Ipomoea* petal development. *J. Exp. Bot.* 61 709–719. 10.1093/jxb/erp335 19933319 PMC2814104

[B64] Yang Q. Yuan D. Shi L. Capell T. Bai C. Wen N. (2012). Functional characterization of the *Gentiana lutea* zeaxanthin epoxidase (GlZEP) promoter in transgenic tomato plants. *Transgenic Res.* 21 1043–1056. 10.1007/s11248-012-9591-5 22297392

[B65] Yuan H. Zhang J. Nageswaran D. Li L. (2015). Carotenoid metabolism and regulation in horticultural crops. *Hortic. Res.* 2:15036. 10.1038/hortres.2015.36 26504578 PMC4591682

[B66] Yuan W. J. Han Y. J. Dong M. F. Shang F. D. (2011). Assessment of genetic diversity and relationships among *Osmanthus fragrans* cultivars using AFLP markers. *Electron. J. Biotechnol.* 14 1–9. 10.2225/vol14-issue1-fulltext-9

[B67] Yuan Y.-W. Sagawa J. M. Young R. C. Christensen B. J. Jr Bradshaw H. D. (2013). Genetic dissection of a major anthocyanin QTL contributing to pollinator-mediated reproductive isolation between sister species of *Mimulus*. *Genetics* 194 255–263. 10.1534/genetics.112.146852 23335333 PMC3632473

[B68] Zhang B. Liu C. Wang Y. Yao X. Wang F. Wu J. (2015). Disruption of a *CAROTENOID CLEAVAGE DIOXYGENASE* 4 gene converts flower colour from white to yellow in *Brassica* species. *New Phytol.* 206 1513–1526. 10.1111/nph.13335 25690717

[B69] Zhang C. Wang Y. Fu J. Bao Z. Zhao H. (2016). Transcriptomic analysis and carotenogenic gene expression related to petal coloration in *Osmanthus fragrans* ‘Yanhong Gui’. *Trees* 30 1207–1223. 10.1007/s00468-016-1359-8

[B70] Zhu C. Bai C. Sanahuja G. Yuan D. Farré G. Naqvi S. (2010). The regulation of carotenoid pigmentation in flowers. *Arch. Biochem. Biophys.* 504 132–141. 10.1016/j.abb.2010.07.028 20688043

[B71] Zhu C. Yang Q. Ni X. Bai C. Sheng Y. Shi L. (2014). Cloning and functional analysis of the promoters that upregulate carotenogenic gene expression during flower development in *Gentiana lutea*. *Physiol. Plant.* 150 493–504. 10.1111/ppl.12129 24256196

[B72] Zhu F. Luo T. Liu C. Wang Y. Yang H. Yang H. (2017). An R2R3-MYB transcription factor represses the transformation of α- and β-branch carotenoids by negatively regulating expression of CrBCH2 and CrNCED5 in flavedo of *Citrus reticulate*. *New Phytol.* 216 178–192. 10.1111/nph.14684 28681945

