# Supplementary material for: Carotenoid Accumulation and Its Contribution to Flower Coloration of Osmanthus fragrans
Source: Front Plant Sci. 2018 Oct 16;9:1499. doi: 10.3389/fpls.2018.01499 (PMC6232703; doi:10.3389/fpls.2018.01499)
Supplement: Supplementary file 1 [file Table_1.pdf]

Supplementary Material

Carotenoid accumulation and its contribution to flower coloration of *Osmanthus fragrans*

Yiguang Wang[1], Chao Zhang[1*], Bin Dong[1], Jianxin Fu[1], Shaoqing Hu[2], Hongbo Zhao[1*]

[1]Department of Ornamental Horticulture, School of Landscape Architecture, Zhejiang Agriculture and Forestry University, Hangzhou, China

[2]College of Civil Engineering and Architecture, Zhejiang Sci-Tech University, Hangzhou, China

***Correspondence:**
Chao Zhang
zhangchao24804@gmail.com
Hongbo Zhao
zhaohb@zafu.edu.cn

Supplementary Tables

Table S1. Primers for carotenoid metaboic genes and reference gene in *O. fragrance* petals.

Gene	Forward primer sequence (5′-3′)	Reverse primer sequence (5′-3′)
OfPSY1	CGCCTGAAGCATTGGATCGTTGG	TTCCAGAGGTCCATCCGCATTCC
OfPDS1	TATCCTCATCGTTTCGTAATTCTCC	TCTCATACCAGTCTCCATCATCATC
OfZ-ISO1	GCCAACCTTCGGTCTCCATTCCAG	TTGGGCAGGTACTTTGGTGTGTGG
OfZDS1	CAGCTTCGTTATAATGGTTGGGTA	GAAAAATCTGCGTCTGGTGTGTAG
OfCRTISO	GAAAAACAAGGGATTCTCGGA	GCAGATAGTAGGCAAGGGTCAA
OfLCYE1	AATTATGGTGTGTGGGAAGATGAG	CAGCACACCTCTTCAGTAACTCCTC
OfLCYB1	GAAAGGAGACGCCAAAGGGAG	GGAAGAAATAGCCGAGATGATAAGA
OfCHYE1	CATCCGCCTGTCCTGTTGAGAAG	ATCTTGACCAGCATTGACCTTGTAGTT
OfCHYB2	ACGAGTCGCACCATAAACCA	CCAAACACCGTAATCCCAAG
OfZEP1	GATGGGGCATTTTTTGTAACTG	ATGGATGAAAACGAACAGGGAA
OfVDE1	CTGAACATAAGGAGCAAGCCAT	CCGCTGAAATACTCCATACCAC
OfNSY1	ACACCACTTTATCACAAGGCGTG	AAATCTGTAACATTCCCTGGTGC
OfNCED3	TTTCAACGAATGTGACGAGGGAT	GGCAAGATACGCATAGCGGGTTT
OfCCD1-1	TGGACAAGATTGCTAAGGTAACTG	TCCAGGTCCCAGATCAAAGATAC
OfCCD1-2	GCGGCATCCTGGTATCACATCTGAA	TGGGCAGTTCGACAACAGCAACA
OfCCD4-1	ACTTTTGTTGACCCTCCTCTTCG	TGACATATCGGCTACAAAGAGTGG
OfACT	CCCAAGGCAAACAGAGAAAAAAT	ACCCCATCACCAGAATCAAGAA

Table S2. Content of carotenoid components in petals of the 24 *O. fragrans* cultivars at full flowering stage.

Sample NO.	Content (μg/g, DW)					
	**c1**	**c2**	**c3**	**c4**	**c5**	**c6**
A1	8.11±0.33	2.48±0.70	1.88±1.61	2.76±1.71	9.16±0.44	14.93±0.53
A2	3.74±0.05	5.57±0.19	0.45±0.13	1.70±0.18	5.66±0.04	26.43±0.09
A3	10.63±0.55	1.78±0.11	0.52±0.07	0.57±0.10	4.99±0.09	9.51±0.18
A4	11.81±0.30	2.23±0.15	1.55±0.61	2.13±0.04	22.09±0.04	55.98±0.27
A5	8.74±0.16	1.46±0.10	-	0.68±0.07	0.96±0.15	5.38±0.06
A6	5.40±0.80	1.58±0.03	1.18 ± 0.10	1.59±0.06	5.24±0.02	57.01±0.25
B1	10.54±0.22	1.63±0.15	1.51 ± 0.42	1.55±0.12	9.28±0.11	32.87±0.16
B2	6.20±0.15	1.47±0.12	0.82±0.30	0.57±0.13	5.39±0.19	18.64±0.18
B3	12.77±0.08	1.81±0.24	1.22±0.12	1.79±0.13	11.79±0.07	28.79±0.25
B4	7.99±0.63	1.05±0.09	0.44±0.00	0.31±0.04	2.37±0.04	8.12±0.14
C1	39.16±4.63	22.14±0.09	97.79±10.29	81.43±9.90	1963.50±192.20	3166.68±302.84
C2	44.30±1.08	22.43±0.47	119.07±1.05	87.70±0.84	2382.77±9.18	7720.69±40.09
C3	36.26±0.81	16.28±0.33	67.64±0.40	62.90±0.28	1136.86±4.10	1988.12±5.57
C4	40.58±0.08	21.31±0.17	109.18±0.75	84.05±0.29	2178.81±9.75	6737.60±30.43
C5	40.52±0.29	21.06±0.14	95.17±0.42	80.84±0.09	1967.61±4.25	5645.12±16.83
C6	41.77±1.36	20.86±0.79	101.26±0.74	75.26±1.35	1622.72±3.97	4602.97±20.44
C7	28.49±1.70	11.99±0.25	75.62±0.66	51.49±0.93	842.61±3.27	1380.08±8.23
C8	13.79±0.21	3.65±0.46	11.70±1.07	11.84±1.21	128.67±1.02	369.95±3.53
D1	9.34±0.16	1.63±0.14	-	0.71±0.02	3.84±0.05	11.60±0.07
D2	9.29±0.19	1.31±0.03	-	0.37±0.00	1.70±0.13	10.65±0.07
D3	7.69±0.19	0.95±0.07	0.30±0.01	0.78±0.32	2.06±0.18	9.28±0.15
D4	9.59±0.56	1.16+0.05	2.07±0.19	1.44±0.04	4.36±0.10	46.46±0.20
D5	8.43±0.06	0.83±0.08	0.51±0.12	0.83±0.12	7.58±0.15	13.83±0.14
D6	10.18±0.08	1.33±0.05	0.54±0.04	0.76±0.02	3.03±0.21	9.18±0.12
