# Supplementary material for: Carotenoid Accumulation and Its Contribution to Flower Coloration of Osmanthus fragrans
Source: Front Plant Sci. 2018 Oct 16;9:1499. doi: 10.3389/fpls.2018.01499 (PMC6232703; doi:10.3389/fpls.2018.01499)
Supplement: Supplementary file 2 [file Image_1.pdf]

Supplementary Material

Carotenoid accumulation and its contribution to flower coloration of *Osmanthus fragrans*

Yiguang Wang[1], Chao Zhang[1*], Bin Dong[1], Jianxin Fu[1], Shaoqing Hu[2], Hongbo Zhao[1*]

[1]Department of Ornamental Horticulture, School of Landscape Architecture, Zhejiang Agriculture and Forestry University, Hangzhou, China

[2]College of Civil Engineering and Architecture, Zhejiang Sci-Tech University, Hangzhou, China

***Correspondence:**
Chao Zhang
zhangchao24804@gmail.com
Hongbo Zhao
zhaohb@zafu.edu.cn

Supplementary Figures

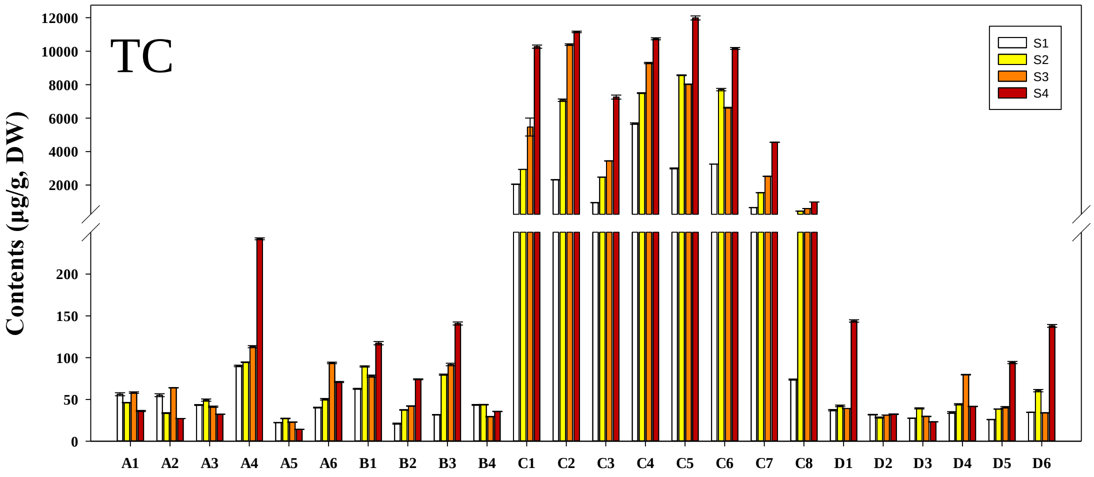

Figure S1. Total carotenoid contents in petals of the 24 cultivars during flowering process.

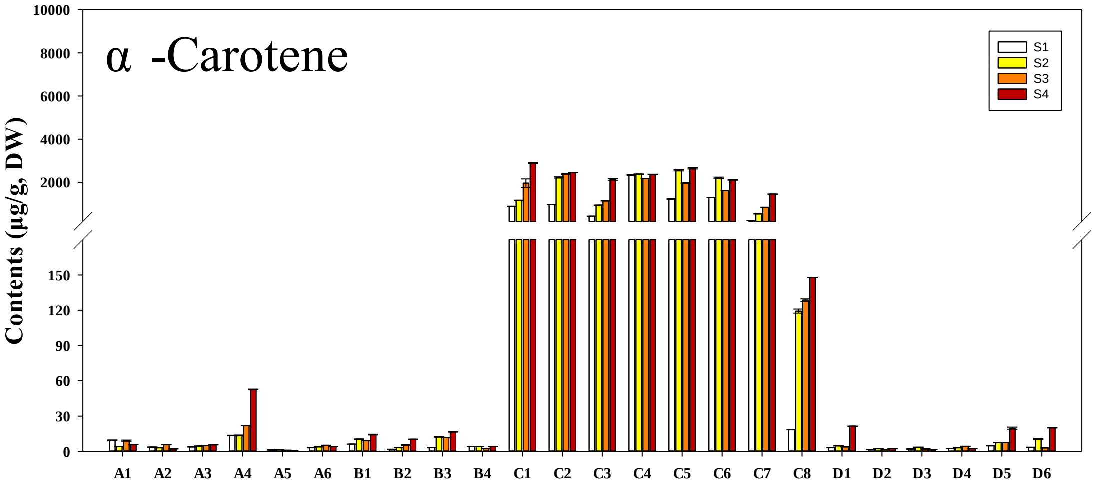

Figure S2. Content of α-carotene in petals of the 24 cultivars during flowering process.

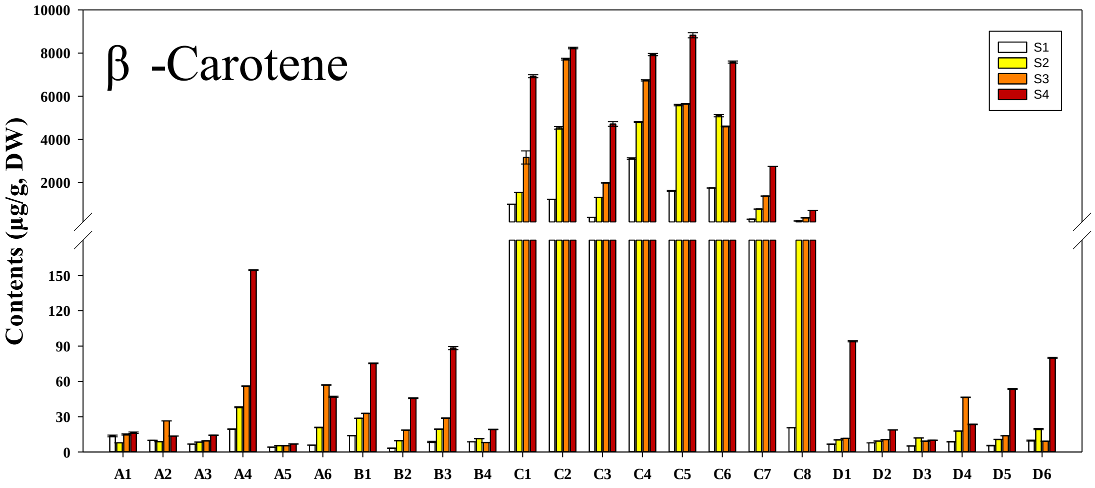

Figure S3. Content of β-carotene in petals of the 24 cultivars during flowering process.

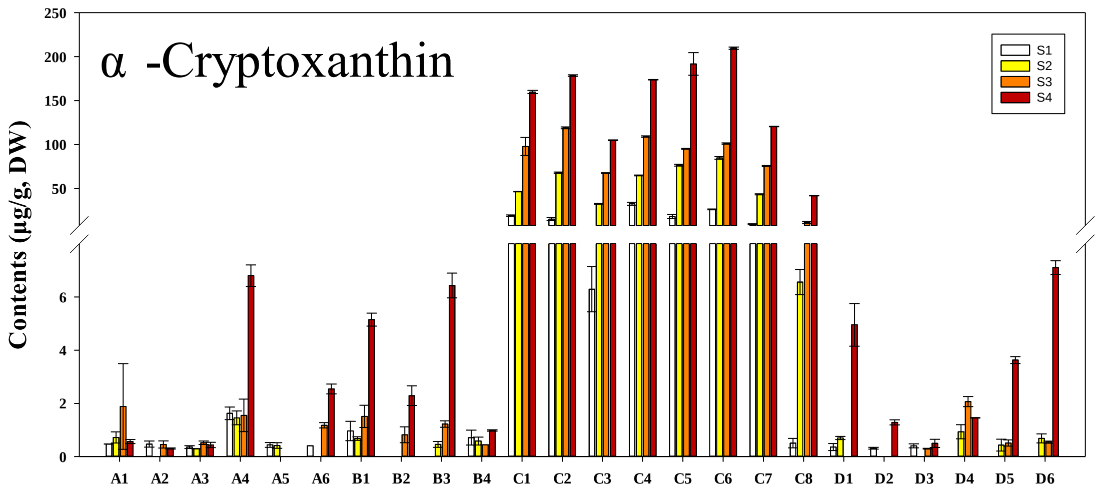

Figure S4. Content of α-cryptoxanthin in petals of the 24 cultivars during flowering process.

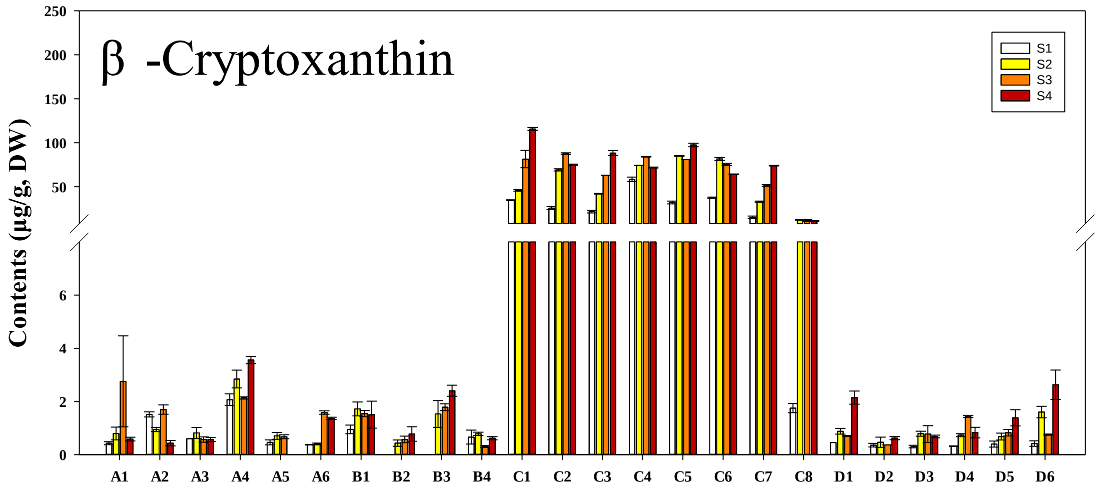

Figure S5. Content of β-cryptoxanthin in petals of the 24 cultivars during flowering process.

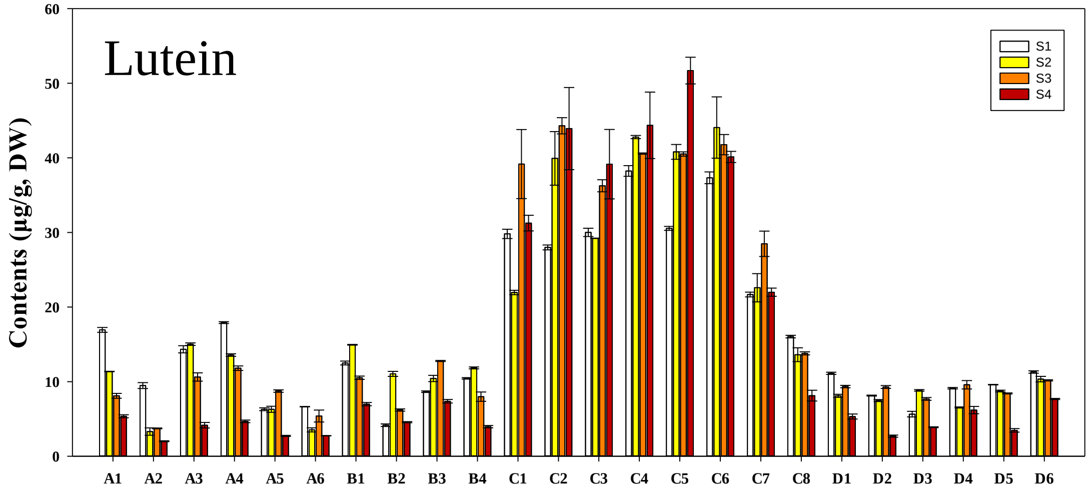

Figure S6. Content of lutein in petals of the 24 cultivars during flowering process.

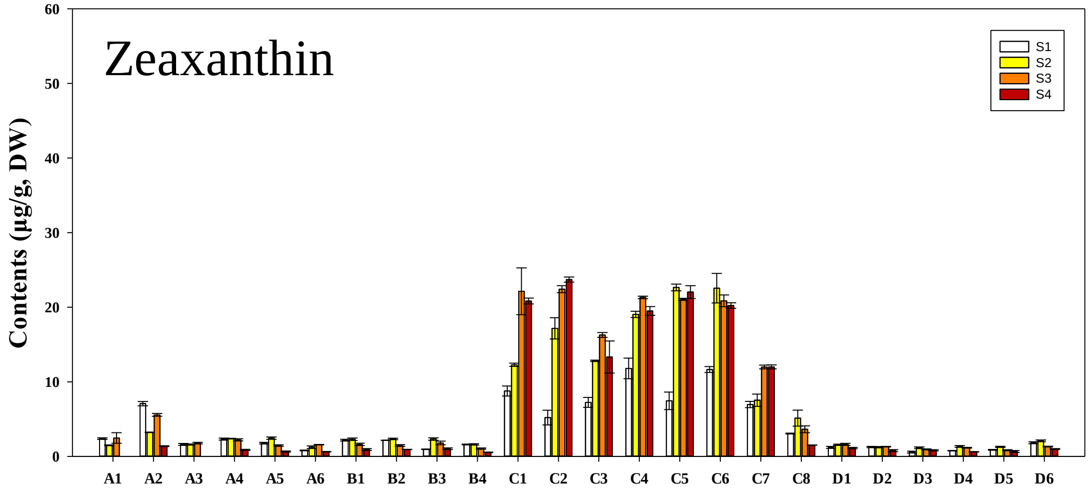

Figure S7. Content of zeaxanthin in petals of the 24 cultivars during flowering process.

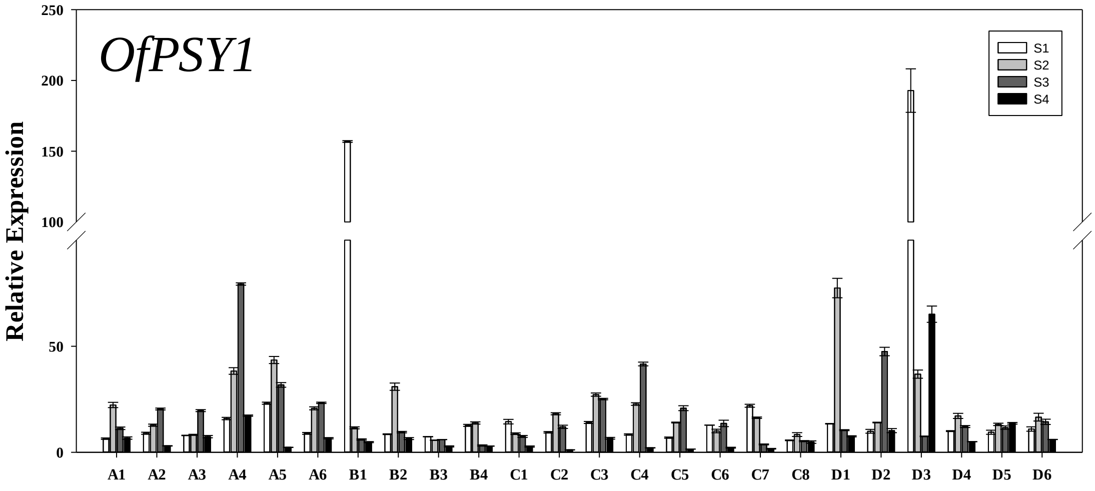

Figure S8. Expression pattern of *OfPSY1* in petals of the 24 cultivars during the flowering process.

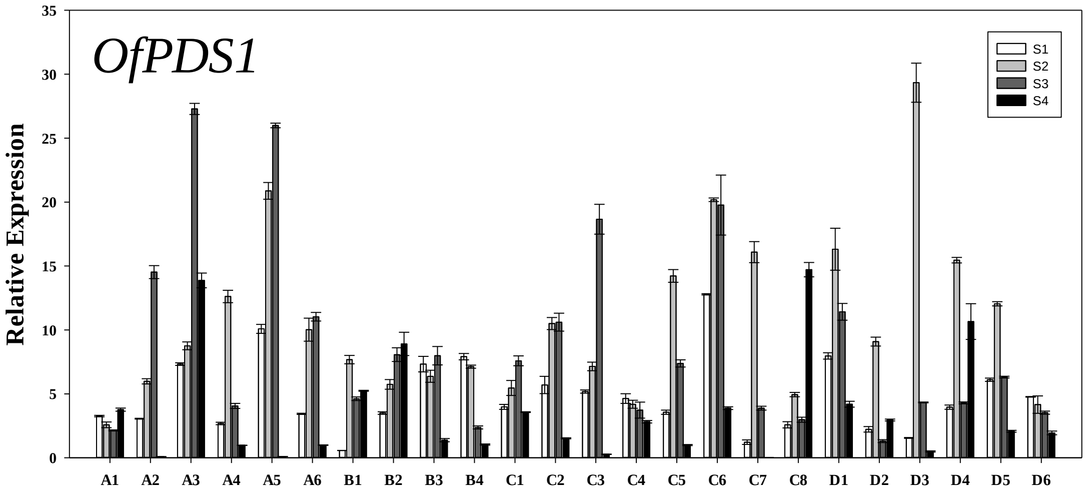

Figure S9. Expression pattern of *OfPDS1* in petals of the 24 cultivars during the flowering process.

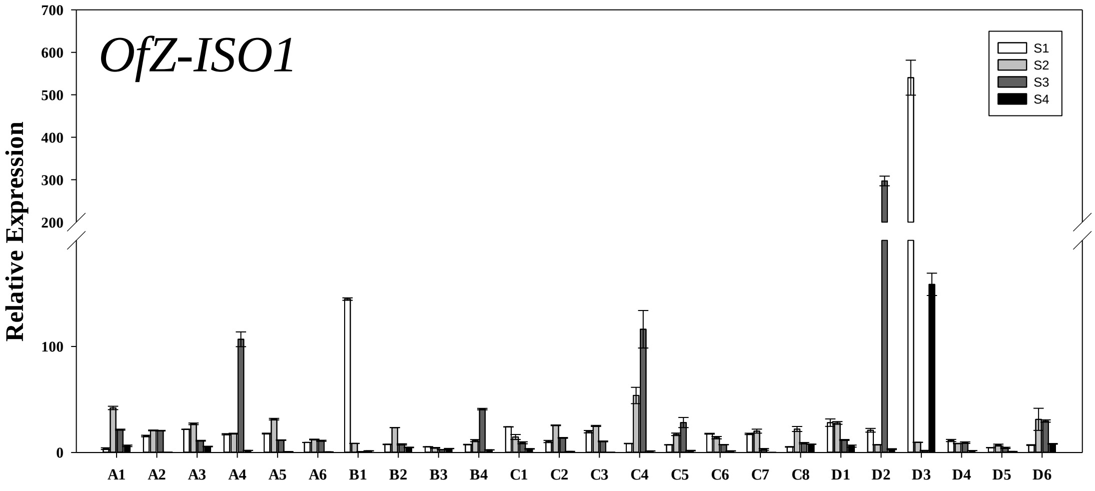

Figure S10. Expression pattern of *OfZ-ISO1* in petals of the 24 cultivars during the flowering process.

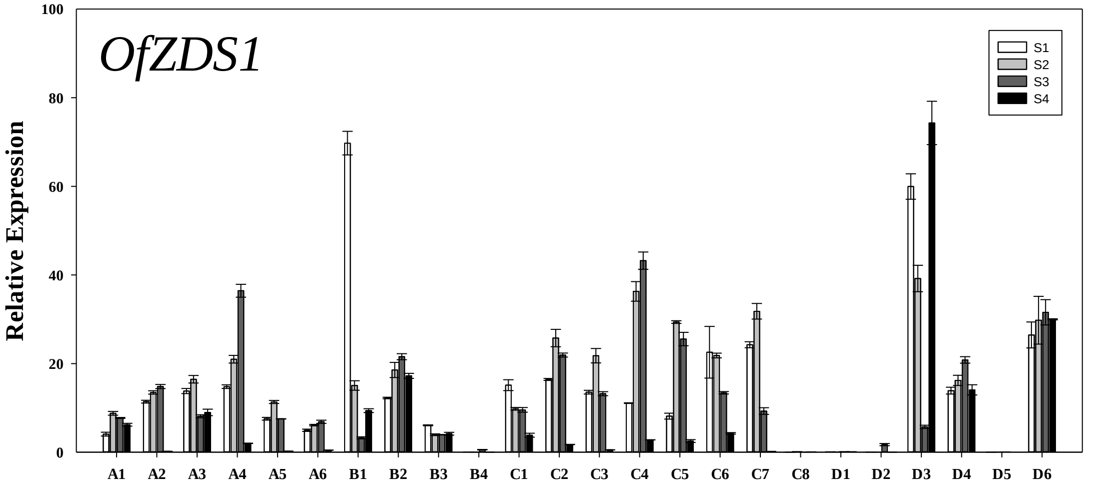

Figure S11. Expression pattern of *OfZDS1* in petals of the 24 cultivars during the flowering process.

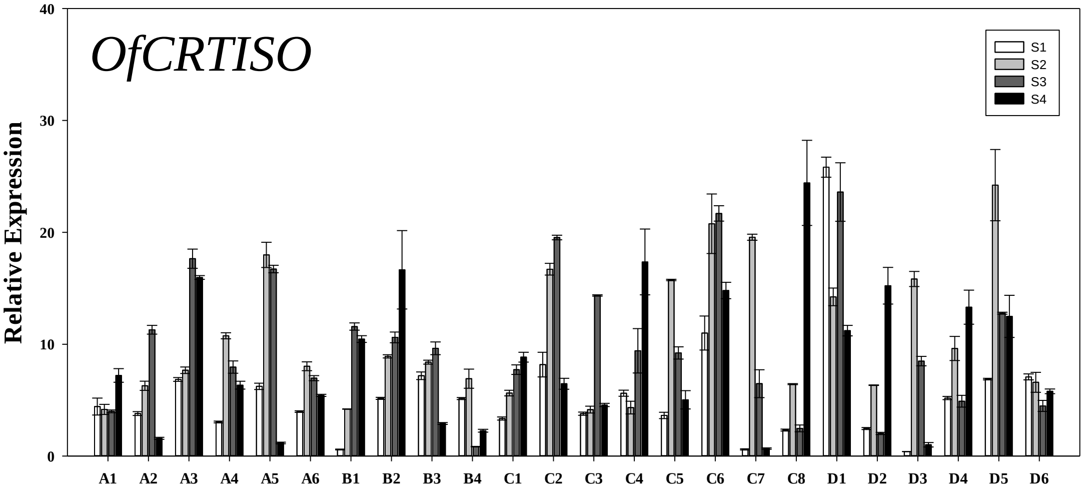

Figure S12. Expression pattern of *OfCRTISO1* in petals of the 24 cultivars during the flowering process.

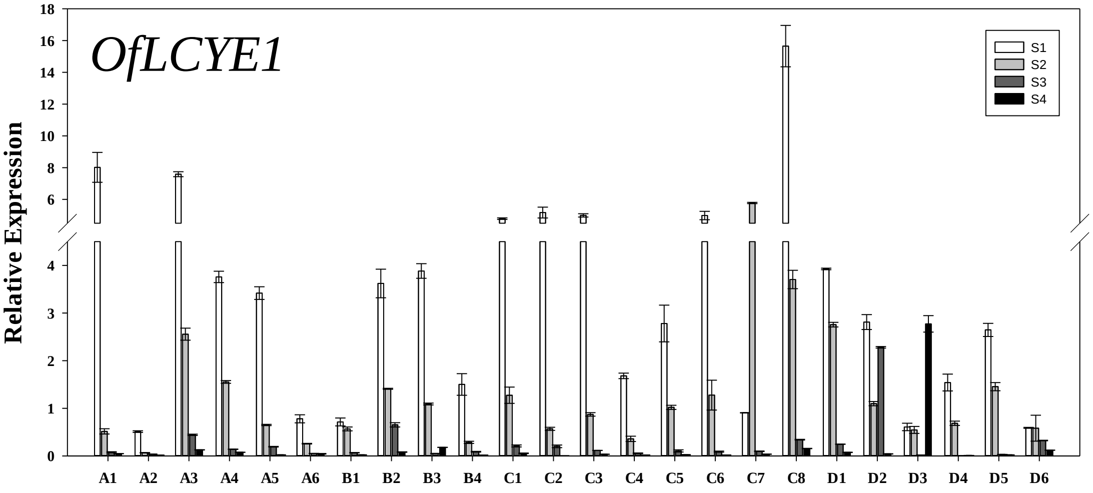

Figure S13. Expression pattern of *OfLCYE1* in petals of the 24 cultivars during the flowering process.

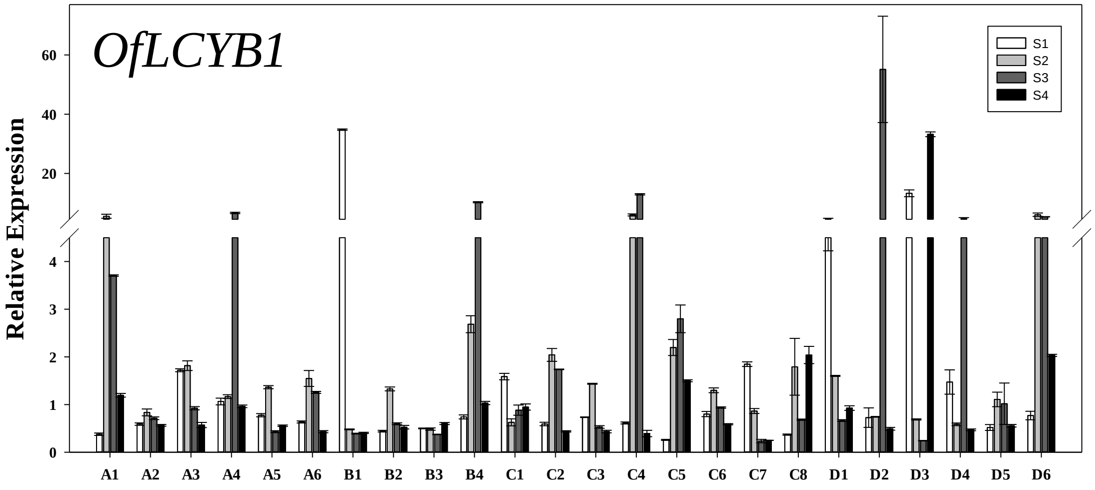

Figure S14. Expression pattern of *OfLCYB1* in petals of the 24 cultivars during the flowering process.

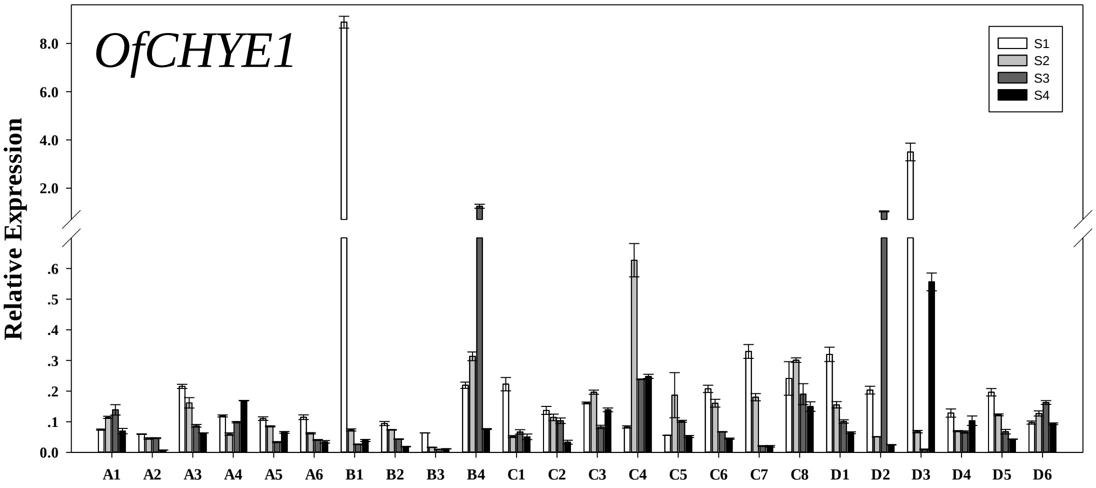

Figure S15. Expression pattern of *OfCHYE1* in petals of the 24 cultivars during the flowering process.

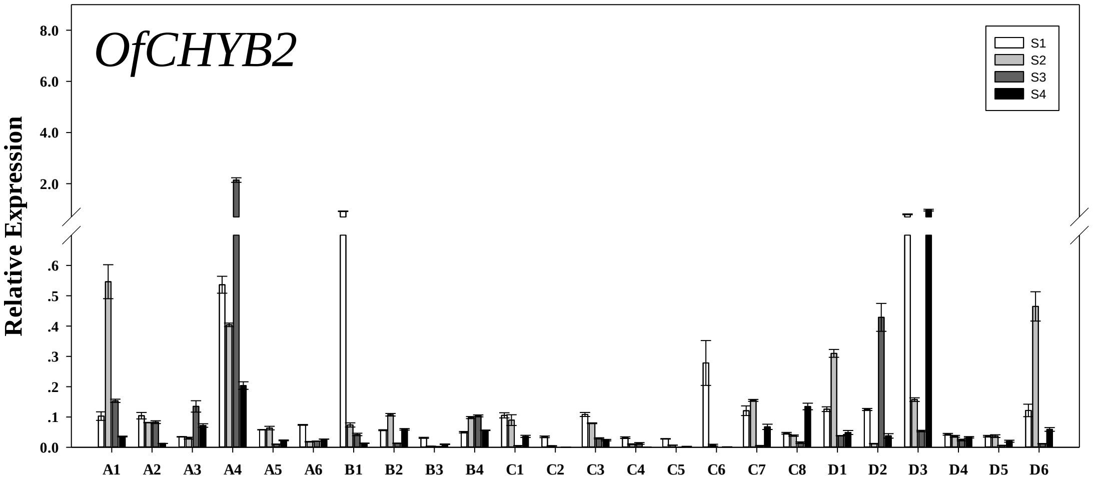

Figure S16. Expression pattern of *OfCHYB2* in petals of the 24 cultivars during the flowering process.

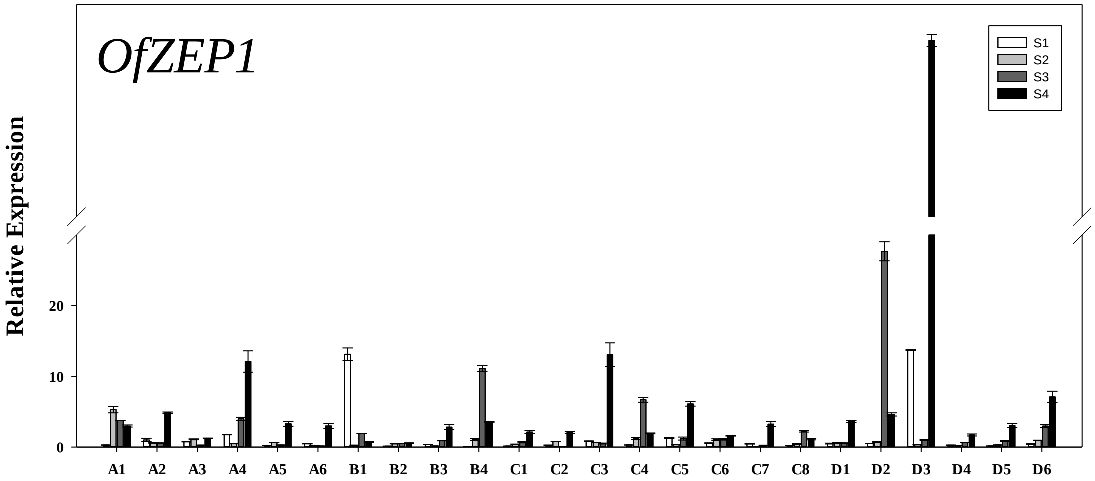

Figure S17. Expression pattern of *OfZEP1* in petals of the 24 cultivars during the flowering process.

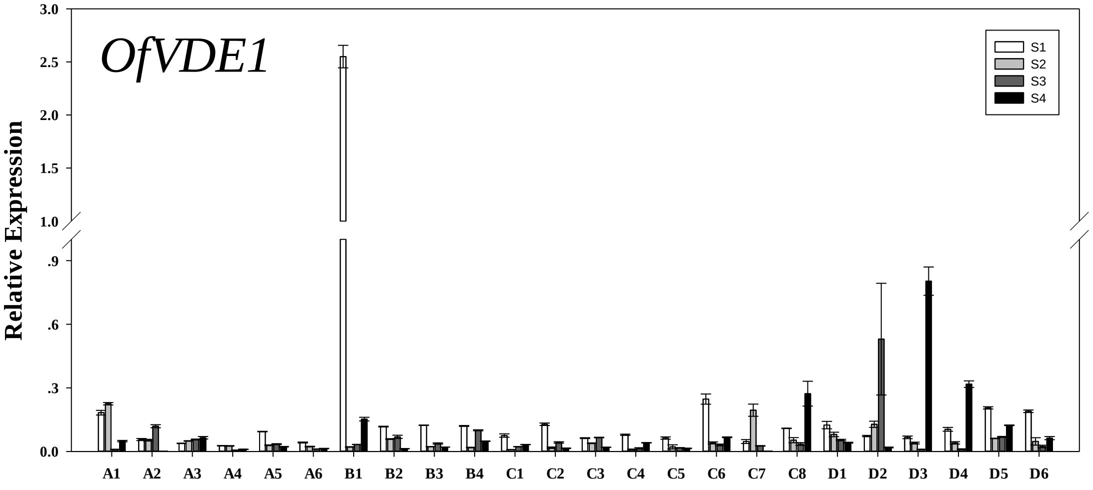

Figure S18. Expression pattern of *OfVDE1* in petals of the 24 cultivars during the flowering process.

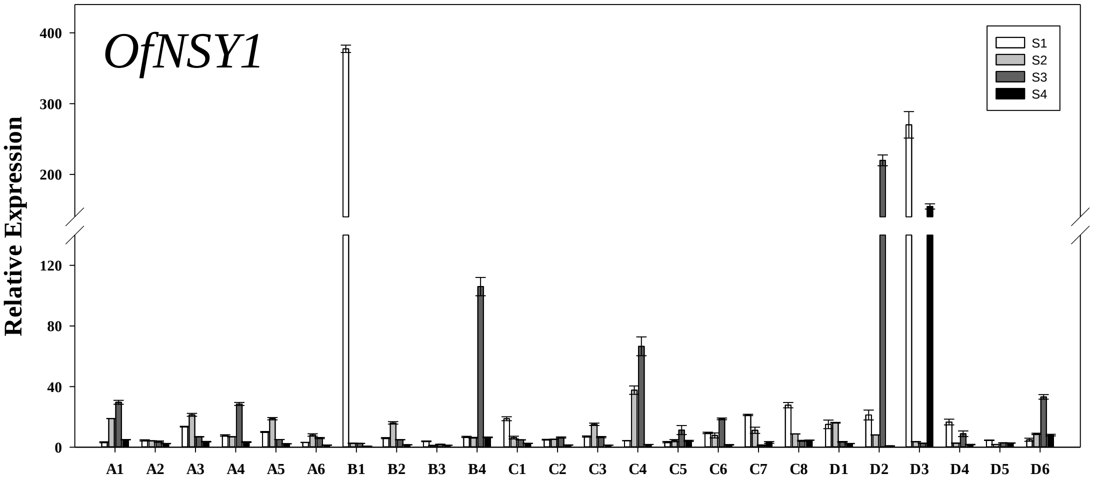

Figure S19. Expression pattern of *OfNSY1* in petals of the 24 cultivars during the flowering process.

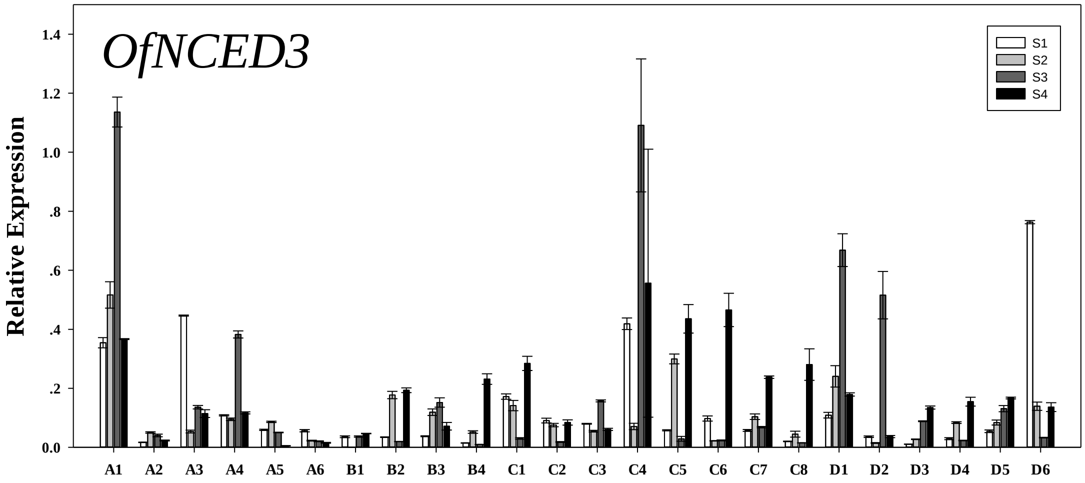

Figure S20. Expression pattern of *OfNCED3* in petals of the 24 cultivars during the flowering process.

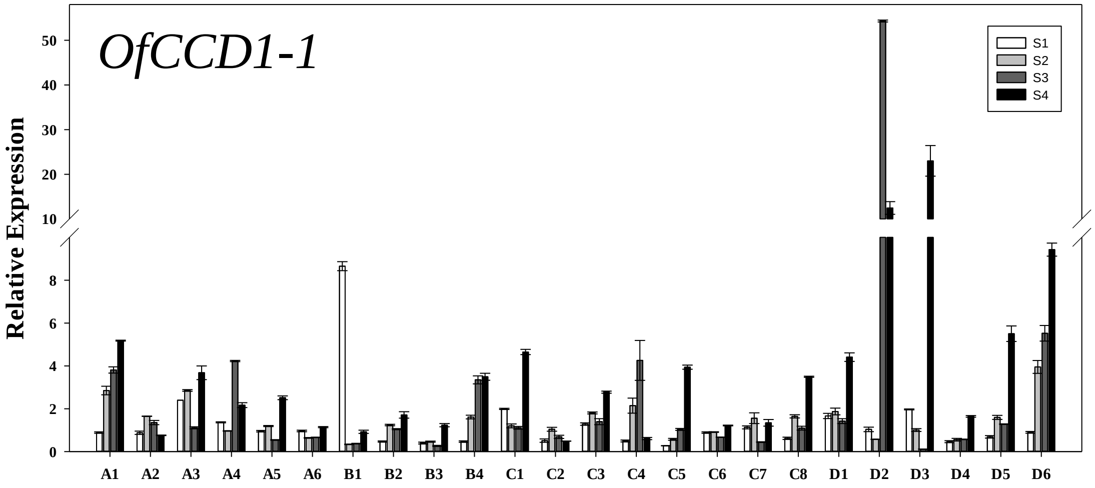

Figure S21. Expression pattern of *OfCCD1-1* in petals of the 24 cultivars during the flowering process.

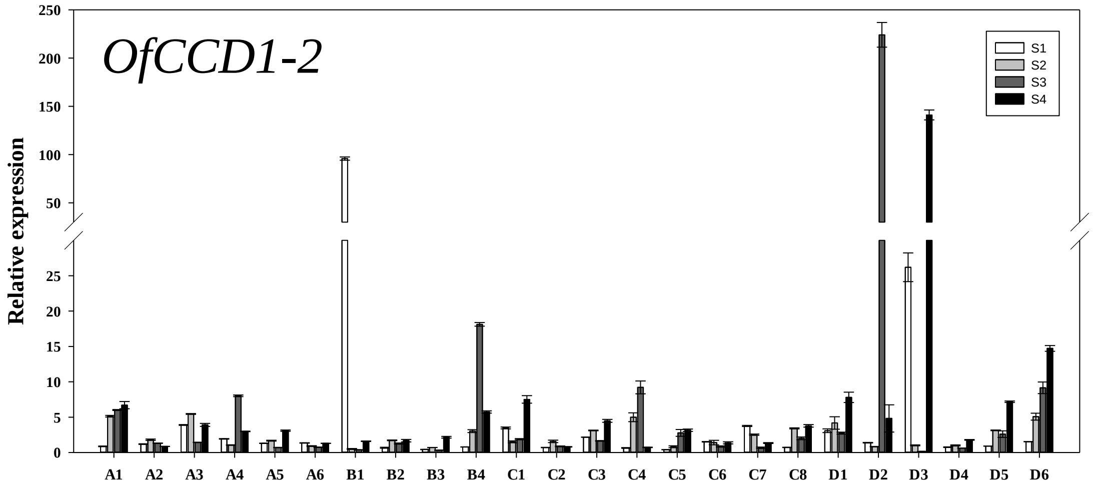

Figure S22. Expression pattern of *OfCCD1-2* in petals of the 24 cultivars during the flowering process.

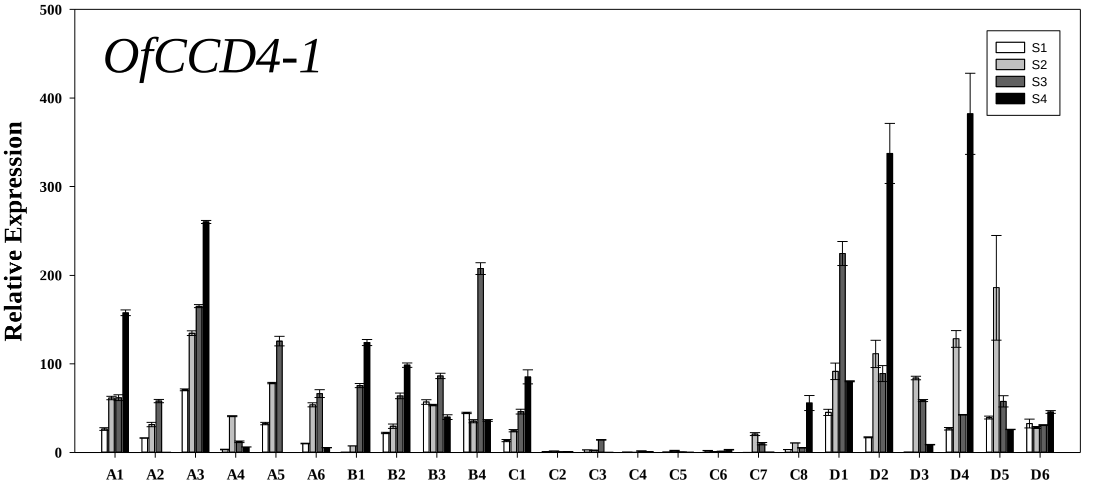

Figure S23. Expression pattern of *OfCCD4-1* in petals of the 24 cultivars during the flowering process.

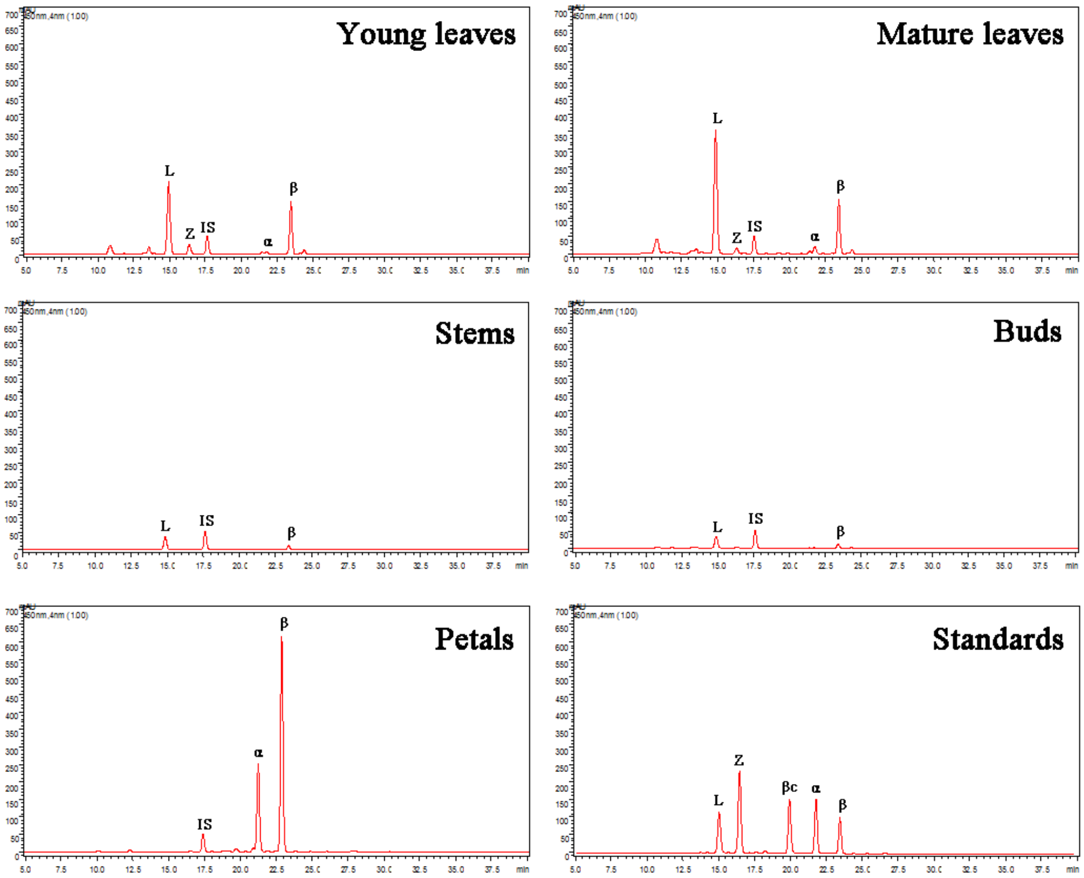

Figure S24. The HPLC chromatogram of carotenoids in the young leaves, mature leaves, stems, buds and petals of *O. fragrans* 'Yan Honggui', and carotenoid standards obtained at 450 nm. L, lutin; Z, zeaxanthin; βc, β-cryptoxanthin; α, α-carotene; β, β-carotene; IS, internal standard.
